# AIM1 is an actin-binding protein that suppresses cell migration and micrometastatic dissemination

**DOI:** 10.1038/s41467-017-00084-8

**Published:** 2017-07-26

**Authors:** Michael C. Haffner, David M. Esopi, Alcides Chaux, Meltem Gürel, Susmita Ghosh, Ajay M. Vaghasia, Harrison Tsai, Kunhwa Kim, Nicole Castagna, Hong Lam, Jessica Hicks, Nicolas Wyhs, Debika Biswal Shinohara, Paula J. Hurley, Brian W. Simons, Edward M. Schaeffer, Tamara L. Lotan, William B. Isaacs, George J. Netto, Angelo M. De Marzo, William G. Nelson, Steven S. An, Srinivasan Yegnasubramanian

**Affiliations:** 1 Sidney Kimmel Comprehensive Cancer Center, Johns Hopkins University, Baltimore, MD USA; 2 Department of Pathology, Johns Hopkins University, Baltimore, MD USA; 3 Department of Environmental Health and Engineering, Bloomberg School of Public Health, Johns Hopkins University, Baltimore, MD USA; 4 Brady Urological Institute, School of Medicine, Johns Hopkins University, Baltimore, MD USA; 5 Department of Chemical and Biomolecular Engineering, Johns Hopkins University, Baltimore, MD USA; 6 Johns Hopkins Physical Sciences in Oncology Center, Johns Hopkins University, Baltimore, MD USA; 7 Office of Scientific Research, Norte University, Asunción,, Paraguay

## Abstract

A defining hallmark of primary and metastatic cancers is the migration and invasion of malignant cells. These invasive properties involve altered dynamics of the cytoskeleton and one of its major structural components β-actin. Here we identify AIM1 (absent in melanoma 1) as an actin-binding protein that suppresses pro-invasive properties in benign prostate epithelium. Depletion of AIM1 in prostate epithelial cells increases cytoskeletal remodeling, intracellular traction forces, cell migration and invasion, and anchorage-independent growth. In addition, decreased AIM1 expression results in increased metastatic dissemination in vivo. AIM1 strongly associates with the actin cytoskeleton in prostate epithelial cells in normal tissues, but not in prostate cancers. In addition to a mislocalization of AIM1 from the actin cytoskeleton in invasive cancers, advanced prostate cancers often harbor AIM1 deletion and reduced expression. These findings implicate AIM1 as a key suppressor of invasive phenotypes that becomes dysregulated in primary and metastatic prostate cancer.

## Abstract (web summary)

Invasion of malignant cells involves changes in cytoskeleton dynamics. Here the authors identify absent in melanoma 1 as an actin binding protein and show that it regulates cytoskeletal remodeling and cell migration in prostate epithelial cells, acting as a metastatic suppressor in cancer cells.

## Introduction

A defining characteristic of carcinoma is cell migration and invasion, first through surrounding tissue architectural confines to form locally invasive lesions, and then through blood and lymphatic vessels and distant tissues during the formation of metastases. Alterations in the dynamics of the actin cytoskeleton, which is critical in determining cell shape and motility, have been implicated in cancer cell migration and tumor progression^1–5^. The actin cytoskeleton is a dynamic cellular scaffold that undergoes constant remodeling to facilitate structural plasticity and regulate cell motility, migration, and invasion^3^. Such remodeling relies on the ability of actin to form filamentous structures by polymerizing actin monomers (G-actin) into actin filaments (F-actin), allowing dynamic regulation of the biomechanical properties of the cell^6^.

Human cancers, including prostate cancer, frequently show morphological and molecular evidence of a dysregulated actin cytoskeleton. Prostate cancer tissues show a higher level of G-actin as compared to normal prostatic epithelium and the actin cytoskeleton frequently appears disorganized in prostate carcinoma^5, 7^. Furthermore, recent in silico meta-analyses of large-scale expression data sets from normal prostate and prostate cancer demonstrate that genes involved in actin cytoskeleton regulation are differentially expressed between tumor and normal tissue in prostate cancer^8–10^. Taken together, these findings strongly suggest that the actin cytoskeleton is profoundly dysregulated in prostate cancer. However, the molecular alterations involved in the dysregulation of the actin cytoskeleton, and their underlying genetic and epigenetic basis are incompletely understood.

Copy-number loss of chromosome 6q12-22 occurs in nearly 30% of primary prostate cancers, and is even more frequent in metastases^11–14^. The core deleted region spans more than 40 Mbp, harboring multiple putative tumor suppressor genes^11^ including *AIM1* (absent in melanoma 1). *AIM1* was initially identified as a putative tumor suppressor using a subtraction cloning approach in a melanoma cell line^15^. Recent studies have also suggested that *AIM1* loss can be mediated by promoter hypermethylation^16, 17^. Structural analysis suggested that AIM1 shows similarity to the superfamily of βγ-crystallin proteins that make up the major structural component of the human lens^18^. However, the role of AIM1 in normal cellular homeostasis and cancer is not established and the molecular functions of AIM1 are unknown.

Here, we show that AIM1 associates with the actin cytoskeleton and suppresses cytoskeletal remodeling and invasive properties in non-malignant prostate epithelial cells. In human prostate cancer tissues, AIM1 dissociates from the actin cytoskeleton. This phenomenon mimics stages of embryonic prostate development in which prostatic buds from the urogenital sinus invade into the surrounding mesenchyme. In more aggressive and metastatic prostate cancers, this mislocalization of AIM1 was compounded by reduced expression and genomic loss. In vivo models further showed that loss of AIM1 led to increased micrometastases of prostate cancer xenografts. These findings suggest that AIM1 is an important regulator of actin cytoskeletal dynamics, cell migration and invasion, and metastatic dissemination in prostate cancer.

## Results

### AIM1 is a β-actin interacting protein

**Fig. 1:**
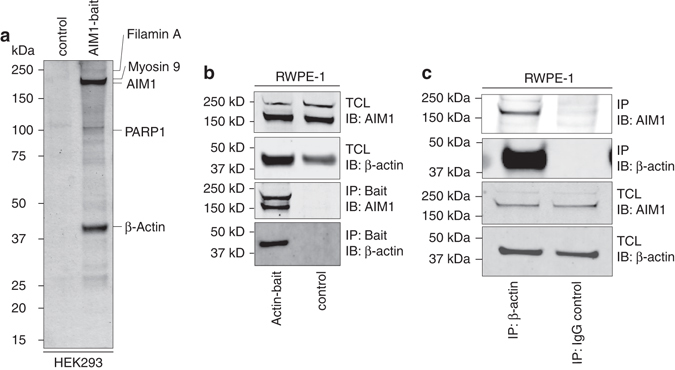
AIM1 interacts with the actin cytoskeleton. Lysates from HEK293 cells transiently transfected with control or biotin-affinity-tagged AIM1 vectors from a single experiment were enriched by streptavidin beads. Co-precipitating complexes were eluted and eluates were separated by PAGE or analyzed by mass-spectrometry. **a** Representative image of a silver-stained gel of control and AIM1 biochemical enrichment experiments. Note the strong enrichment of a 42 kDa protein representing β-actin. **b** Endogenous AIM1 interacts with affinity-tagged β-actin in normal prostate epithelial cells (RWPE-1). Representative gel of three independent experiments is shown. **c** Pulldown of endogenous β-actin shows robust co-precipitation of AIM1 in RWPE-1 cells. Two percent of TCL was loaded as control. *IB* immunoblot, *IP* immunoprecipitate, *TCL* total cell lysate

Since the function of AIM1 was unknown, we first conducted an unbiased proteomic interaction screen by overexpressing affinity-tagged AIM1 in HEK293 cells. Bead-based affinity-enrichment followed by mass-spectrometry of AIM1 and control vector expressing cells revealed 79 (FDR < 1%) interacting proteins in a single experiment (Fig. 1a, Tables 1 and 2, Supplementary Table 1). A strong enrichment for components of the actin cytoskeleton, in particular β-actin, non-muscle myosin 9, and filamin A, was observed; additionally, gene set enrichment analysis of the interacting proteins demonstrated a predominance of proteins involved in actin-based movement and cytoskeletal organization (Tables 1 and 2, Supplementary Table 1). This is of particular importance, since expression changes in gene sets involved in actin cytoskeletal regulation are among the most common alterations observed in prostate cancers (Supplementary Fig. 1). These results demonstrate that AIM1 can associate with endogenous β-actin in HEK293 cells. To confirm this interaction in a reciprocal manner in prostate epithelial cells, we overexpressed affinity-tagged β-actin in RWPE-1 non-malignant prostate epithelial cells and probed β-actin bait-specific precipitates with custom-made rabbit antibodies against AIM1 (Fig. 1b), showing that AIM1 co-precipitates with β-actin. To further establish that β-actin and AIM1 interact at endogenous expression levels, we immunoprecipitated cell lysates from RWPE-1 cells with β-actin-specific antibodies and probed the precipitate with AIM1-specific antibodies. We found robust co-immunoprecipitation of AIM1 with β-actin-specific antibodies, providing further evidence that both proteins form a complex under physiological expression levels in non-malignant prostate epithelial cells (Fig. 1c).

**Table 1:** Top 20 co-precipitating proteins

Protein ID	Molecular weight (kDa)
Absent in melanoma 1	189
Myosin, heavy polypeptide 9	227
Beta actin	42
Myosin, heavy polypeptide 10	229
Cardiac muscle alpha actin 1	42
Vimentin	54
Keratin 2	65
Heat shock 70 kDa protein 1B	70
Keratin 6A	60
Keratin 1	66
Clathrin heavy chain 1	192
Drebrin 1 isoform a	71
Keratin 5	62
Keratin 10	59
Nucleolin	77
Filamin A, alpha isoform 1	280
Poly (ADP-ribose) polymerase	113
Tubulin, beta	50
Calmodulin 2	17
Keratin 14	52

**Table 2:** Functional gene-set analysis (GO) of co-precipitated proteins

GO biological process term	FDR
Translational elongation	0.0011
Actin filament-based process	0.0049
Intracellular transport	0.0060
Actin filament-based movement	0.0076
Cytoskeleton organization	0.0159

### The C terminus of AIM1 is required for β-actin interaction

**Fig. 2:**
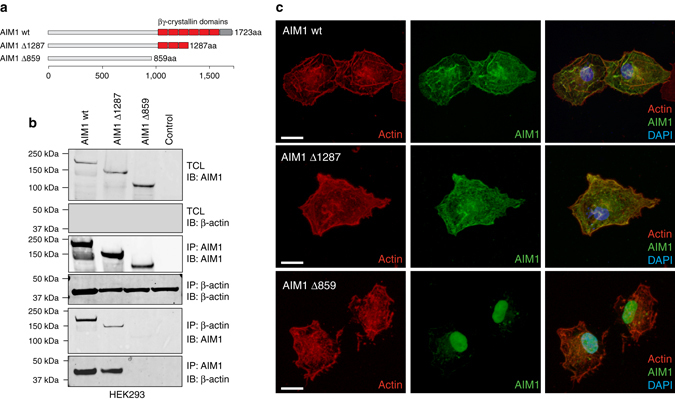
The C terminus of AIM1 is required for the efficient interaction of AIM1 with the actin cytoskeleton. **a** Ideogram depicting the domain structure of AIM1 and the C-terminal deletion constructs used in the study (wt, denotes wild-type AIM1; Δ1287, indicates C-terminal deletion to amino acid 1287; Δ859, indicates C-terminal deletion to amino acid 859). **b** Reciprocal co-immunoprecipitations of wt AIM1 and AIM1 deletion mutants in HEK293 cells demonstrate that the Δ859 C-terminal deletion mutant, lacking all βγ-crystallin domains, fails to interact with the actin cytoskeleton, while full length and Δ1287 AIM1 retain binding to the actin cytoskeleton. Immunoblots shown here are representative of three independent experiments. **c** Confocal immunofluorescence microscopy demonstrates strong co-localization of actin (visualized by alexa-568-labeled phalloidin, shown in *red*) with YFP-tagged (shown in *green*) wt and Δ1287 AIM1 in transiently transfected COS7 cells. Scale bars indicate 5 μm. AIM1 Δ859 shows a predominant nuclear localization and no co-localization with actin. Two percent of TCL was loaded as control. *IB* immunoblot, *IP* immunoprecipitate, *TCL* total cell lysate

To determine the protein domains required for the interaction between AIM1 and β-actin, we generated C-terminal deletion constructs of AIM1 (Fig. 2a) and tested their ability to interact with β-actin in co-precipitation experiments in HEK293 cells, as described above. Whereas wild-type AIM1 (wt) and truncation mutant Δ1287 showed reciprocal co-immunoprecipitation with β-actin, the truncation mutant Δ859, which is devoid of the entire βγ-crystallin domain repeat, did not (Fig. 2b). These results suggest that βγ-crystallin domain structures are necessary for complex formation. This hypothesis was further corroborated using immunofluorescence microcopy in COS7 cells overexpressing different AIM1 YFP-tagged truncation constructs. Wild-type as well as Δ1287 showed a cytoplasmic staining pattern with a strong co-localization with β-actin (Fig. 2c). Strikingly, the Δ859 mutant showed no co-localization with β-actin in the cytoplasm and mostly nuclear accumulation. These observations suggest that the C-terminal βγ-crystallin domains of AIM1 are required for the interaction with the actin cytoskeleton.

### AIM1 depletion results in enhanced cytoskeletal remodeling

**Fig. 3:**
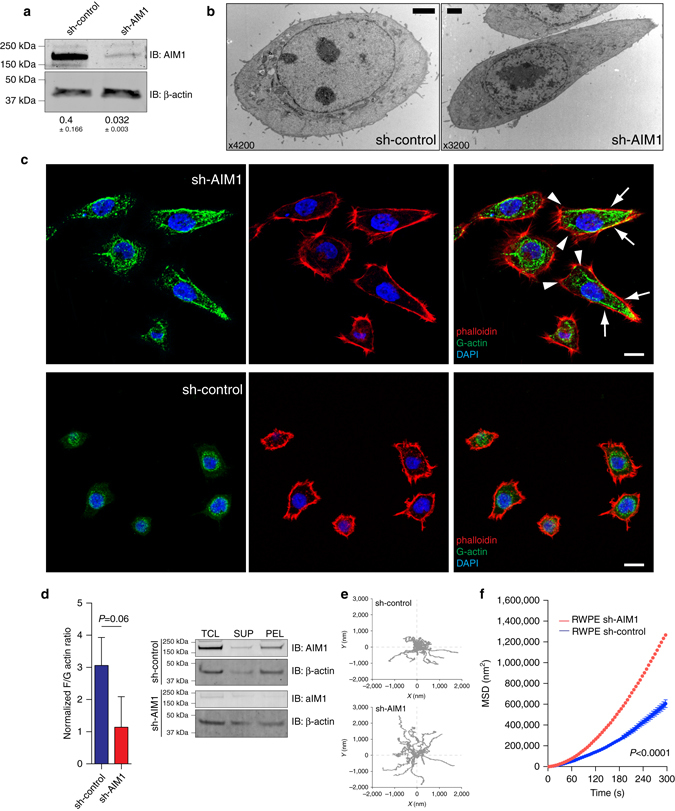
AIM1 depletion results in increased formation of cell protrusions and enhanced cytoskeletal remodeling. **a** Western blot confirming AIM1 knockdown in sh-AIM1 cells. Numbers below blots show normalized AIM1 intensities (±SD) from three independent experiments. **b** Electron microscopic ultrastructural analyses reveal increased cell size and formation of broad cell protrusions at the leading edge in RWPE-1 cells depleted of AIM1. Scale bar indicates 2 μm. **c** Immunofluorescence microscopy using probes specific to F-actin (AlexaFluor-labeled phalloidin, *red*) and G-actin (monoclonal G-actin-specific antibody, *green* ^21^) in AIM1 proficient and deficient RWPE-1 cells further corroborate the increased size and cell protrusion formation phenotype in sh-AIM1 cells and demonstrate an increased accumulation of G-actin in the trailing edge of sh-AIM1 cells (*arrowheads* indicate broad cell protrusions at the leading edge, *arrows* indicate trailing edge). Scale bar indicates 10 μm. **d** Biochemical fractionation of G-actin and F-actin by differential centrifugation reveals increased abundance of G-actin in sh-AIM1 cells. PEL pellet after ultracentrifugation (containing F-actin pool), SUP supernatant after ultracentrifugation (containing G-actin pool), TCL total cell lysate. *Left panel* shows densitometric quantification of three independent biochemical fractionation experiments, representing the mean ± SD. **e** Representative bead trajectory plots for sh-control and sh-AIM1 cells selected from three independent experiments. **f** Mean-squared displacements (MSD) of nanoscale bead motion analysis in RWPE-1 sh-AIM1 and sh-control cells shows substantially increased nanoscale motion suggestive of increased cytoskeletal remodeling in RWPE-1 sh-AIM1 cells (*P* < 0.0001) (*t*-test *P* values)

To better understand the role of AIM1 in cell biology, we generated benign prostate epithelial cells (RWPE-1) overexpressing shRNAs targeting AIM1 (sh-AIM1) or control shRNA (sh-control) (Fig. 3a) and evaluated cell morphological differences by transmission electron microscopy (TEM). Interestingly, we found that AIM1-depleted cells appeared significantly larger and showed increased broad protrusions located at the leading edge of cells (Fig. 3b and Supplementary Fig. 2)^19, 20^. Under these culture conditions, no differences in filopodia formation were observed.

β-actin is present in two states in the cell: as a globular monomer (G-actin) or as a filamentous polymer (F-actin). Previous studies have suggested that invasive carcinomas show increased cellular G-actin^5^, which was associated with invasive cancer cell phenotypes. Given the association of AIM1 with β-actin, we assessed whether AIM1 modulation could alter the levels of F-actin and G-actin. We first used confocal immunofluorescence microscopy to elucidate the organization of F-actin and G-actin in situ in RWPE-1. Using the chemical probe phalloidin, which binds to filamentous actin, and a G-actin-specific antibody, we showed that cells depleted of AIM1 exhibited increased cellular G-actin. Importantly G-actin was localized in the trailing edge of the AIM1-depleted cells (Fig. 3c). In addition, we performed biochemical fractionation of F-actin and G-actin by ultracentrifuge sedimentation, wherein F-actin can be found precipitated in the pellet, while G-actin can be detected in the supernatant. SDS-PAGE followed by antibody-mediated detection of β-actin in three independent experiments showed a trend for decreased F-actin/G-actin ratios in AIM1-depleted cells (Fig. 3d). Probing cell lysates from sh-control and sh-AIM1 cells with antibodies specific to G-actin also showed that AIM1-depleted cells had significantly higher levels of G-actin (Supplementary Fig. 2)^21^.

Since increased G-actin is associated with increased cytoskeletal remodeling, we next assessed the molecular-biomechanical consequences of AIM1 perturbation on cytoskeletal dynamics. Using our sh-control and sh-AIM1 RWPE1-1 cells, we directly visualized the real-time, spontaneous, nanoscale tracer motions (SNTMs) of functionalized microbeads attached to the cytoskeleton through cell surface integrin receptors (Supplementary Fig. 2), as described previously^22, 23^. The SNTM were characterized as the mean-squared displacements (MSDs) across all microbeads as a function of time (Fig. 3e, f). Both AIM1-expressing and AIM1-silenced RWPE-1 cells exhibited MSD that increased with time as a power law with an exponent *α* > 1, indicating an underlying super-diffusive and active process attributable to molecular-level remodeling of the actin cytoskeleton, and not Brownian motion^23^. However, compared with sh-control cells, AIM1-depleted cells exhibited marked increases in SNTM (Fig. 3e), with increased MSD that became apparent at times >20 s, with continual increases through 300 s (Fig. 3f, Supplementary Fig. 2F–I). In addition, AIM1-depleted cells showed marked increases in the super-diffusive exponent *α* (1.49 + 0.04 vs. 1.69 + 0.06; *P* < 0.05, unpaired *t*-test; Supplementary Fig. 2). We found similar changes with two additional lentiviral shRNAs targeting AIM1 (Supplementary Fig. 2). These observations indicate that AIM1 depletion alters cytoskeletal remodeling and is associated with changes in cellular G-actin and F-actin pools.

### AIM1 modulates cellular traction forces and FAK signaling

**Fig. 4:**
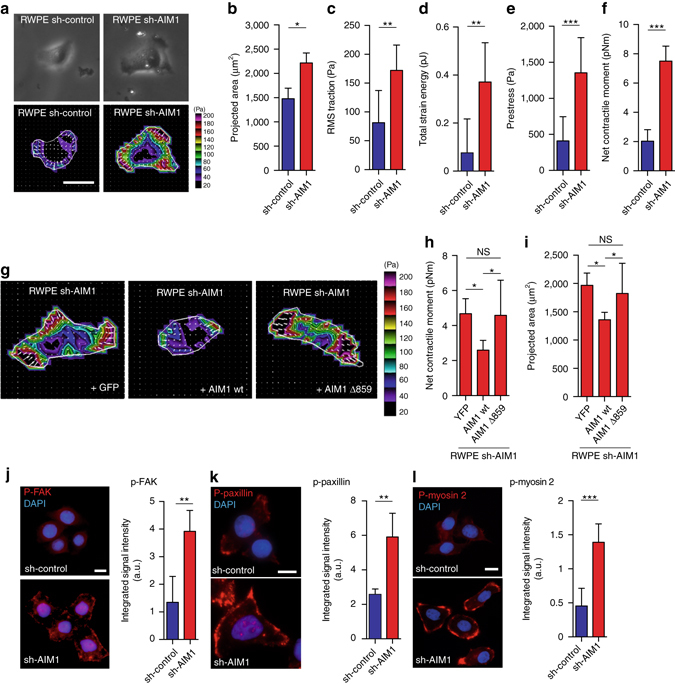
AIM1 depletion increases cytoskeletal remodeling, cellular traction forces and focal adhesions in prostate epithelial cells. **a** Representative images of phase contrast and Fourier transform traction microscopy (FTTM) for RWPE-1 sh-AIM1 and sh-control cells, show the magnitude (*colorscale*) and direction (*arrows*) of traction forces. Scale bars indicate 10 μm. From these FTTM experiments, projected area (**b**), root-mean-squared (RMS) traction force (**c**), total strain energy (**d**) prestress force (**e**), and net contractile moment (**f**) were determined, showing significant increases in sh-AIM1 compared to sh-control RWPE-1 cells for all measures. **g**–**i** From traction force vectors (**g**), the calculated net contractile moments (**h**), and projected cellular area (**i**) of RWPE-1 sh-AIM1 “rescued” with YFP control, wild-type AIM1 or AIM1 Δ859 are shown. Note that expression of wild-type AIM1 can rescue the increased contractile moment and projected area in RWPE-1 sh-AIM1 cells. **j**–**l** Immunofluorescence microscopy experiments revealed that levels of phosphorylated focal adhesion kinase (p-FAK) (**j**), phosphorylated paxillin (p-paxillin) (**k**), and phosphorylated myosin 2 (p-myosin 2) (**l**), were significantly increased in sh-AIM1 compared to sh-control RWPE-1 cells, indicating an increase of focal adhesions in sh-AIM1 cells. **a**, **g**, **j**–**l** All microscopy images are representative of two to three independent experiments. Scale bars indicate 5 μm. All bar plots represent mean ± SD of 20–50 single cells from each experiment. *T*-test *P* values, * denotes *P* < 0.05; ** denotes *P* < 0.01; *** denotes *P* < 0.001

The cell’s ability to exert force upon its surrounding matrix is an important determinant of cellular migratory and invasive properties^24, 25^. To better determine the role of AIM1 in regulating physical forces, we measured the traction stress-generation of individual AIM1-proficient and AIM1-deficient cells using Fourier transform traction microscopy (FTTM)^26^. In this technique, we measured, in 2D, the deformation field arising at the interface between each adherent cell and the precisely tuned elastic matrix on which it is attached. Using the deformation field and known elastic material properties of the matrix, we could explicitly map the corresponding intracellular traction field (Fig. 4a). Compared with AIM1-expressing cells, AIM1-depleted cells were larger in size (Fig. 4a, b) and exercised greater traction (root mean square) averaged over the entire cell-projected area (Fig. 4c). From the computed traction, we also derived a number of other physical measures^26^, including the strain energy imparted by the cell to the substrate (Fig. 4d), the tensional stress borne by actin microfilaments (Fig. 4e), and the amplitude of the cell’s contractile strength, the net contractile moment (Fig. 4f). All computed physical metrics of forces were significantly greater in AIM1-depleted cells than in AIM1-expressing cells (Fig. 4). Most interestingly, overexpression of full-length AIM1, but not AIM1 Δ859 in AIM1-deficient cells rescued the alterations in cell morphology and the cell’s contractile strength (Fig. 4g–i). Collectively, these cellular biomechanical experiments suggest that AIM1 plays an important role in suppressing actin cytoskeletal remodeling dynamics and force-generating capacity.

The generation of forces on the underlying surface is dependent on cytoskeletal contraction and on the adhesion of the cell to the underlying surface via focal adhesion. Focal adhesion kinase (FAK) is a key regulator in the formation of focal adhesions. We therefore evaluated FAK activation in AIM1-depleted and control cells by investigating the phosphorylation status of tyrosines 925 and 576/577 of FAK, which have previously been shown to be tightly linked to FAK activity^27, 28^. By western blot analyses, we found that both FAK activation-specific phosphorylation marks were greatly increased in AIM1-deficient cells (Supplementary Fig. 2L). In addition, larger and more numerous phospho-FAK (T925) positive focal adhesions were present in sh-AIM1 (Fig. 4j). Next, we determined the level of phospho-paxillin (Tyr118), a well-established mark of focal adhesions and a direct target of FAK^29, 30^. Western blot analysis showed increased levels of phospho-paxillin and immunofluorescence microscopy demonstrated an increase in size and number of phospho-paxillin (Tyr118) associated focal adhesions in cells depleted of AIM1 (Fig. 4k, Supplementary Fig. 2M). This observation suggests that loss of AIM1 increased focal adhesion formation through activation of FAK signaling. Myosin 2 has been shown to play a key role in cell adhesion and cell migration^32^. We therefore evaluated the phosphorylation status of myosin-2 (at Thr18/Ser19) in RWPE-1 cells. AIM1-depleted cells showed a significant increase in myosin-2 phosphorylation, which supports the prior observation of increased traction forces in AIM1-deficient cells (Fig. 4l). Taken together, these findings strongly suggest that AIM1 is involved in inhibiting β-actin dynamics and cytoskeletal remodeling, ultimately suppressing the cell’s ability to exert forces on the surrounding matrix.

### AIM1 depletion increases cell motility and cell invasion

**Fig. 5:**
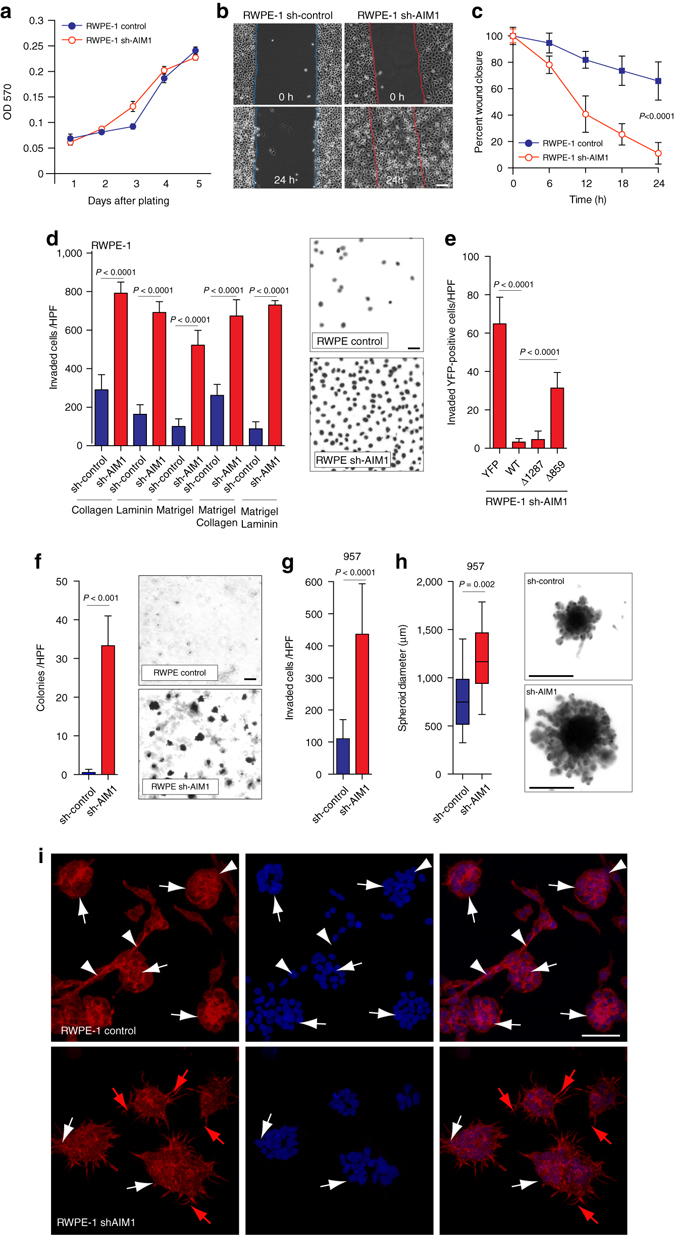
Loss of AIM1 increases cell invasion and cell motility without significantly altering cell proliferation. **a** Knock-down of AIM1 in RWPE-1 cells does not alter cell proliferation. **b** sh-AIM1 and control RWPE-1 cells grown to a monolayer were serum starved for 12 h. After introducing a scratch, cells were cultured in growth medium for 24 h and cell migration was monitored by live cell microscopy (also see Supplementary Movies 1 and 2). Scale bars indicate 100 μm. **c** Quantification of cell migration experiments show robust wound closure of RWPE-1 cells depleted of AIM1 in a 24-h time frame (mean ± SD for five replicates). **d** RWPE-1 cells depleted of AIM1 show dramatically increased cell invasion in Boyden Chamber invasion assays. RWPE-1 sh-AIM1 and control cells were plated in transwells containing isolated extracellular matrix components (laminin and collagen), matrigel or combinations. Forty-eight hours after seeding, invaded cells were quantified (mean ± SD for four replicates). Scale bars indicate 10 μm. **e** This invasion phenotype can be fully rescued in sh-AIM1 RWPE-1 cells by overexpression of full-length AIM1 (WT) and the actin-binding-proficient mutant Δ1287, but not by the actin-binding-deficient mutant AIM1 Δ859 or YFP-control (mean ± SD for four replicates). **f** Knockdown of AIM1 increases anchorage-independent growth of RWPE-1 cells in agarose matrices (mean ± SD for three replicates). Scale bars indicate 100 μm. **g** Depletion of AIM1 increases invasion through a matrigel matrix of prostate epithelial cells 957 (mean ± SD for three replicates). **h** AIM1 depletion in 957 cells results in increased spheroid size in spheroid invasion assays. Note the increased size of spheroidal structures and the increased number in invading protrusions. Scale bar indicates 500 μm (*box-and-whisker plots* of five replicates, with *whiskers* representing the range). All *P* values are derived using *t*-test statistics. **i** RWPE-1 control and shAIM1 cells were grown in matrigel for 5 days. After fixation, cells were stained with AlexaFluor-labeled phalloidin (*red*) and DAPI (*blue*) and imaged by confocal microscopy. Note that RWPE-1 control cells formed defined acinar structures (*white arrows*) with connecting branching structures (*white arrowheads*). This highly organized branching/tubular architecture was disrupted in RWPE-1 shAIM1 cells, which instead showed spindly elongated protrusions (*red arrows*). Scale bars indicate 200 μm

Given the observed effects of AIM1 depletion on actin cytoskeletal dynamics and cellular traction forces, we hypothesized that AIM1 depletion may induce invasive and migratory phenotypes and modulate 3D growth dynamics. Interestingly, while sh-RNA-mediated depletion of AIM1 did not change cell proliferation (Fig. 5a and Supplementary Fig. 3A), AIM1 depletion significantly increased cell motility; AIM1-depleted cells filled scratched wounds completely in a time period of 24 h, whereas sh-control cells only showed minimal motility under these conditions (Fig. 5b, c, Supplementary Movies 1 and 2). In Boyden chamber invasion assays, a significant increase in cell invasion was noted in cells depleted of AIM1 by multiple sh-AIM1 constructs in prostate epithelial cells (RWPE-1, 957) as well as in prostate cancer cell lines (VCaP and PC3) (Fig. 5d and Supplementary Fig. 3). This phenotype was observed using different invasion matrix barriers (collagen, laminin, and matrigel), persisted after controlling for increased cell motility, and was reverted by overexpression of full length AIM1 but not as much by expression of the actin-binding mutant AIM1 Δ859 (Fig. 5e).

An important characteristic of transformed cells is their anchorage-independent growth in soft agar. It has been established previously that RWPE-1 cells do not efficiently form colonies in soft agar^31^. To test whether AIM1 knockdown could increase anchorage-independent growth, sh-AIM1 and sh-control RWPE-1 and 957 cells were plated on agar, and cultured for 14 days. sh-AIM1 cells showed significantly increased anchorage-independent growth on soft agar suggesting that loss of AIM1 can contribute to cell transformation (Fig. 5f, g). However, sh-RNA-mediated stable depletion of AIM1 was not sufficient to render RWPE-1 cells tumorigenic in murine xenografts (Supplementary Fig. 3G). Furthermore, in 3D spheroid basement membrane invasion assays, AIM1 depletion resulted in an increased size of cell spheroids and greatly increased the number of invading cell protrusions, consistent with an increased invasion phenotype (Fig. 5h). In addition, under collagen-enriched 3D matrigel culture conditions, sh-control cells formed highly organized acinar structures with branching architecture as described previously^32^. Interestingly, RWPE-1 cells depleted of AIM1 were characterized by complete absence of branching ductal structures and disrupted and disorganized acinar structures with stellate protrusions (Fig. 5i), similar to spindle-like filopodia seen in comparable 3D culture conditions of other invasive prostate cancer cell lines^33^. Taken together, these data suggest that AIM1 loss can promote disorganized growth of prostate epithelial cells while promoting migratory and invasive phenotypes.

### AIM1 suppresses metastatic dissemination in vivo

**Fig. 6:**
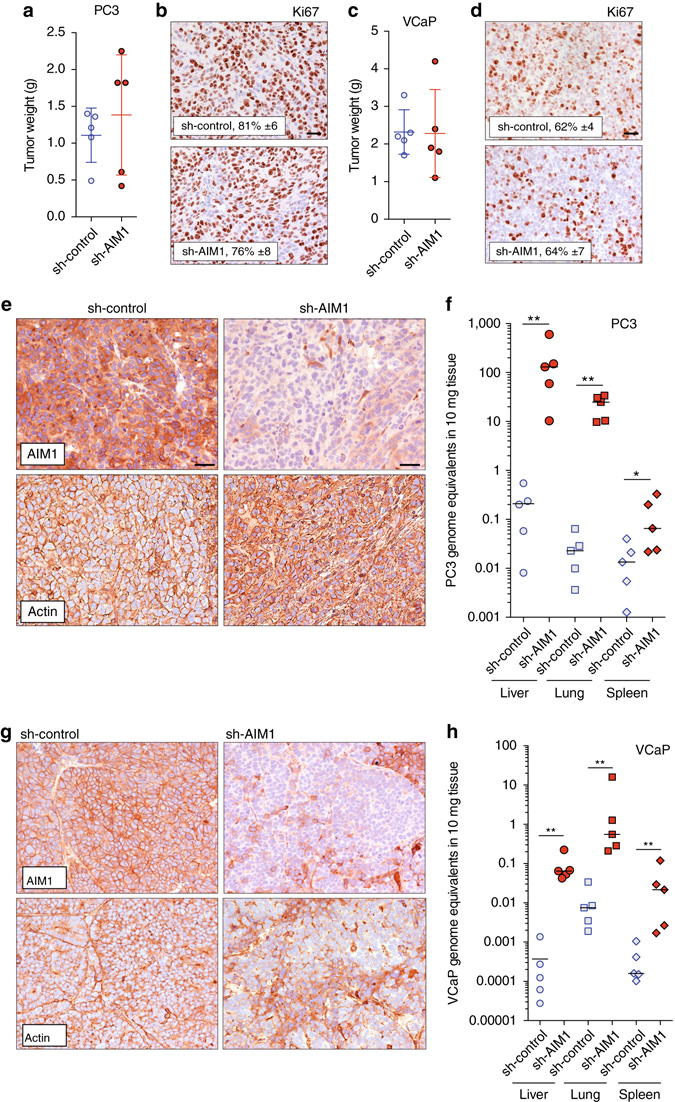
AIM1 depletion results in increased micrometastatic dissemination in vivo. Xenograft tumors derived from sh-control and sh-AIM1 expressing PC3 and VCaP prostate cancer cell lines (five animals per group) were grown in the flank of nude mice for 4 and 6 weeks, respectively. **a**, **c** Tumor weights at the time of necropsy of PC3 and VCaP xenografts. Note that there was no statistically significant difference in end of study tumor weights between sh-control and sh-AIM1 xenografts of PC3 and VCaP cells. **b**, **d** Cell proliferation as determined by Ki67 immunostaining is not different in sh-control and sh-AIM1 xenografts. The percentage of Ki67 positive cells is indicated. Scale bars indicate 50 μm. **e** Representative micrographs of immunostains for AIM1 and actin in PC3 sh-control and sh-AIM1 xenograft tumors demonstrate significant depletion of AIM1 protein levels in sh-AIM1 tumors and increased cytoplasmic staining of actin. Scale bars indicate 50 μm. **f** Micrometastatic burden, measured as PC3 cell equivalents, as determined by *Alu*-specific quantitative PCR in liver, lung and spleen from sh-control and sh-AIM1 PC3 xenograft bearing animals (*n* = 5 in each group). **g** Representative micrographs of immunostains for AIM1 and actin in VCaP sh-control and sh-AIM1 xenograft tumors demonstrate significant depletion of AIM1 protein levels in sh-AIM1 tumors and increased cytoplasmic staining of actin. **h** Micrometastatic burden, measured as VCaP cell equivalents, as determined by *Alu*-specific quantitative PCR in liver, lung and spleen from sh-control and sh-AIM1 bearing animals (*n* = 5 in each group). *P < 0.05, **P < 0.01 (*t*-test *P* values)

To evaluate the role of AIM1 on tumor growth and micrometastatic dissemination in vivo, we generated prostate cancer cell lines (VCaP and PC3) that stably overexpress either AIM1-targeting shRNAs (sh-AIM1) or non-targeting control shRNAs (sh-control) and established tumor xenogafts. There was no statistically significant difference in tumor size or cell proliferation index (as determined by Ki67 immunostaining) in the primary tumor site at the flank for VCaP or PC3 xenografts expressing sh-AIM1 or control constructs (Fig. 6a–d). Immunohistochemical studies confirmed robust depletion of AIM1 protein levels in sh-AIM1 expressing xenograft tumors (Fig. 6e, g). Interestingly, the organization of the actin cytoskeleton appeared disrupted in the AIM1-depleted xenografts from both cell line models (Fig. 6e, g *bottom panels*). To determine the micrometastatic tumor burden, at the time of necropsy, lung, liver, and spleen tissues were harvested from tumor-bearing mice and the DNA copy number (as cancer cell genomic equivalents) of *Alu* DNA elements were determined by quantitative PCR in target tissues. Since the *Alu* repetitive element is not found in murine genomes, but is present at high copy number in human genomes, real-time PCR-based assays determining the *Alu* copy number have been used and validated to quantitate human cancer cells in murine tissues^34^. These analyses revealed that the micrometastatic burden was greatly increased in mice harboring xenografts of AIM1-depleted cells with a median increase for all sites of 194-fold (range 6–8378-fold, *P* < 0.0001) and 426-fold (range 0.59–10,415-fold, *P* < 0.0001) for VCaP and PC3 cells, respectively (Fig. 6f, h). However, at the time of necropsy, there were no overt metastatic tumor foci appreciable by gross or microscopic examination. These findings suggest that AIM1 loss leads to increased micrometastatic dissemination.

### AIM1 dissociates from the actin cytoskeleton in cancer

**Fig. 7:**
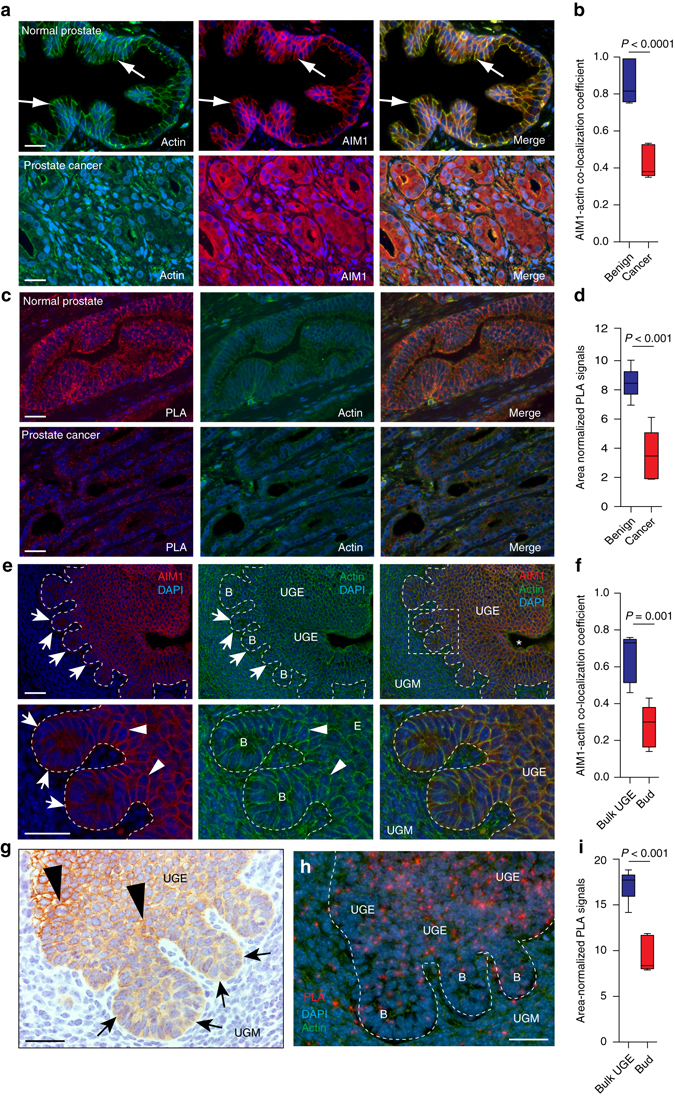
AIM1 and actin localization in normal prostate, prostate adenocarcinoma and prostate embryonic development. **a** Co-immunolabeling of AIM1 (*red*) and β-actin (*green*) reveals high degree of co-localization in normal prostate epithelium. In prostate cancer, this co-localization is largely abolished and AIM1 shows a diffuse cytoplasmic staining pattern. Nuclei are counterstained with DAPI. **b** *Boxplot* shows distribution of co-localization coefficients of AIM1 and β-actin immunostaining in prostate cancer and adjacent benign prostate epithelium (*n* = 8 cases). Scale bars indicate 50 μm. **c** Proximity ligation assays (PLA) confirm close spatial proximity of AIM1 and β-actin in benign prostate epithelial cells and greatly reduced interaction in invasive carcinoma. *Red dots* indicate discrete interaction signals by PLA; *green* shows β-actin immunostaining. Scale bars indicate 100 μm. **d** *Boxplot* shows differences in area-normalized PLA signals in benign glands and invasive carcinoma (*n* = 8 cases). **e** Immunolabeling for Aim1 (shown in *red*) and β-actin (shown in *green*) in urogenital mesenchyme (UGM) and urogenital sinus epithelium (UGE) from mouse embryos at day 17.5 post conception. Note the UGE-specific expression of Aim1, with membranous staining and actin co-localization in bulk UGE (*arrowheads*), and more diffuse cytoplasmic staining in UGE-derived prostatic buds (labeled B) invading into the surrounding UGM (*arrows*). Scale bars indicate 100 μm. **f** *Boxplot* shows distribution of co-localization coefficients of Aim1 and β-actin immunostaining in UGE close to the urogenital sinus (Bulk UGE) and invading prostatic buds (Bud) (*n* = 4). **g** IHC confirms membranous staining pattern of Aim1 in bulk UGE and decreased, diffuse Aim1 immunoreactivity in invading buds. **h** PLA demonstrates spatial proximity of Aim1 and β-actin UGE close to the urogenital sinus and decreased PLA signals in invading prostate buds. Scale bars indicate 100 μm. **i** *Boxplot* shows differences in area-normalized PLA signals in bulk UGE and invading buds (*n* = 3). All *P* values are derived using *t*-test statistics

To evaluate AIM1 protein expression and subcellular localization, we optimized immunohistochemical staining conditions for formalin-fixed paraffin-embedded tissues (FFPE) and validated the specificity of the custom made AIM1 antibodies on genetically controlled cell line models (Supplementary Fig. 4A). We then co-immunolabeled slides containing normal prostatic epithelium as well as high-grade prostate cancer with AIM1 and β-actin-specific antibodies (Fig. 7a). AIM1 was highly expressed in normal prostatic luminal epithelium (Fig. 7a, Supplementary Fig. 4B). In these cells, AIM1 was closely associated with β-actin, both of which showed a membranous staining pattern, with accentuated staining around the apical tight junction complex (*arrows*, Fig. 7a; mean co-localization coefficient = 0.82, SD = 0.11). These data suggested that AIM1 and β-actin are associated in a complex in normal prostate epithelial cells in human tissues. Importantly, in prostatic adenocarcinoma, the high degree of co-localization between AIM1 and β-actin seen in normal prostate tissues was profoundly disrupted (mean co-localization coefficient = 0.38, SD = 0.08), with AIM1 showing a diffuse cytoplasmic distribution and no accentuated membranous staining, and with β-actin showing a disrupted, patchy, membranous, and cytoplasmic localization pattern (Fig. 7a, b).

The β-actin-AIM1 co-localization phenotypes were further corroborated using in situ proximity ligation assay (PLA)^35^. This assay allows the assessment of spatial proximity of two antibody targets and generates a positive signal only when the two target molecules are in close spatial proximity. The assay showed a high specificity with very limited background signal (Supplementary Fig. 4C). A strong signal for AIM1–actin interactions was observed in normal prostate epithelium, whereas invasive carcinoma showed a greatly decreased interaction (Fig. 7c, d). Taken together, this indicates that AIM1 and actin form a complex in normal prostate epithelial cells and that this interaction is largely abolished in prostate cancer.

Cell invasion processes observed in prostate cancers often represent a re-awakening of embryonic developmental and differentiation programs that were active in phases of physiological invasion during organogenesis. Importantly, the investigation of overlapping morphologic and molecular alterations during neoplastic and developmental invasion processes has allowed a refined characterization of key pro-invasive alterations^36, 37^. During prostate development in the urogenital sinus (UGS), prostatic buds form from the urogenital sinus epithelium (UGE) and invade in the surrounding urogenital sinus mesenchyme (UGM) allowing the formation of ductal and acinar structures of the prostate gland. Given the redistribution of AIM1 in invasive prostate cancer, we sought to investigate changes in AIM1 expression during murine prostate development. We isolated developing prostates from mice and found robust AIM1 expression in the UGE during all stages of development (Supplementary Fig. 4D, E). Interestingly, while the UGE showed strong membranous staining for Aim1 co-localizing with actin, the prostatic buds invading into the UGS mesenchyme (at day 17.5 post conception) showed significantly reduced Aim1 peri-membranous staining and greatly decreased co-localization with actin (Fig. 7e–g). This observation was further corroborated by PLA, showing a significant reduction in PLA signals in the invading prostatic buds (Fig. 7h, i). Thus, the dissociation of AIM1 from the actin cytoskeleton seen in invasive prostate cancer tissues resembles an embryonic state involving invasion of prostatic buds into the surrounding mesenchyme during development of the prostate gland.

### AIM1 alterations are associated with aggressive disease

**Fig. 8:**
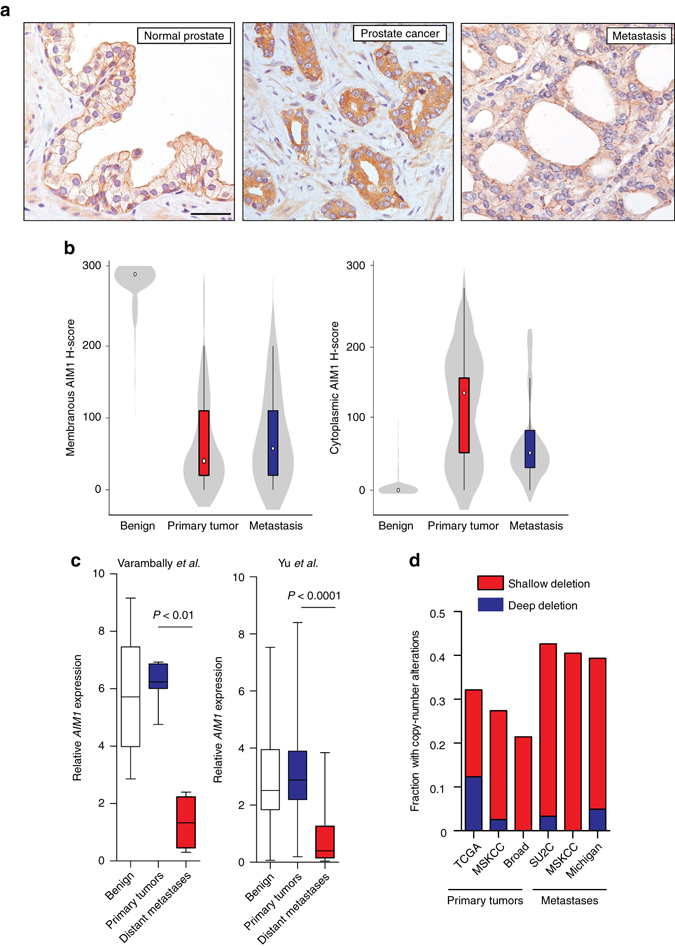
AIM1 mislocalization and reduced expression is associated with clinicopathologic features of prostate cancer. **a** Immunohistochemical analysis of 81 normal, 87 primary tumors, and 52 lymph-node metastases stained with AIM1-specific antibodies. Staining intensities and distributions were scored separately for membranous and cytoplasmic staining patterns using the H-score system. Representative micrographs of AIM1 immunohistochemistry in normal prostate, primary prostate cancer, and prostate metastasis are shown. Note the predominant membranous staining pattern in the normal prostate epithelium in contrast to the diffuse cytoplasmic staining in primary tumor and metastatic lesions. The metastasis also showed overall reduced AIM1 expression compared to primary cancer. Scale bars indicate 100 μm. **b** *Violin plots* show H-score distribution for membranous and cytoplasmic immunostaining in normal prostatic epithelium, primary tumors, and metastases. **c** *AIM1* mRNA expression analysis in two independent cohorts reveals profound *AIM1* downregulation in distant prostate cancer metastases^38, 39^. **d** Frequency of copy-number loss of the AIM1 gene locus from five independent data sets^13, 40, 42–44^

We next assessed whether AIM1 mislocalization and expression levels were associated with prostate cancer clinicopathologic characteristics. We investigated the AIM1 expression pattern in a cohort of 104 patients, from whom 81 morphologically benign prostate sections, 87 sections of prostate adenocarcinoma of various Gleason grades, and 52 lymph-node metastases were examined (Fig. 8a, b, Table 3, Supplementary Fig. 4F). Stained slides were evaluated using the H-score system by which distribution and intensity of membranous and cytoplasmic staining were scored separately. Normal prostate epithelium uniformly showed high membranous and very low cytoplasmic staining for AIM1, whereas adenocarcinoma showed a profoundly decreased membranous staining and increased diffuse cytoplasmic accumulation of AIM1 (Fig. 8a, b, Table 3, Supplementary Fig. 4F). Additionally, we observed a correlation between Gleason grade and membranous AIM1 staining scores, with lower grade lesions (Gleason ≤6) retaining more membranous staining and showing less cytoplasmic accumulation of AIM1 compared to higher-grade lesions (Table 3, Supplementary Fig. 4F, Supplementary Tables 2–4). Interestingly, in metastatic prostate cancer, AIM1 was not only mis-localized to the cytoplasm, but also showed overall reduced protein expression (Fig. 8a, b).

**Table 3:** H-scores of membranous and cytoplasmic AIM1

	Gleason score			
	≤6	7–8	≥9	***P*** value*
*Membranous H-Score*				0.0001
Mean (SD)	235 (105)	125 (111)	131 (112)	
*Cytoplasmic H-Score*				0.0001
Mean (SD)	36 (70)	65 (71)	75 (75)	

SD standard deviation

*Kruskal–Wallis test

To contextualize these protein expression patterns, we examined *AIM1* mRNA expression in prostate cell lines and mined publically available gene expression datasets of prostate tissues. *AIM1* mRNA was expressed in the prostate cell lineage, in normal prostate epithelial cells, and in multiple prostate cancer cell lines independent of their androgen receptor status (Supplementary Fig. 5A–C). Interestingly, in two independent data sets^38–40^, *AIM1* mRNA expression was greatly reduced in metastatic prostate cancer compared to primary cancer or benign prostate (Fig. 8c), consistent with the observation of reduced AIM1 protein expression in metastatic cancers and with a role of AIM1 loss in migration and metastatic dissemination. This notion was further corroborated by the observation that lower relative expression of *AIM1* mRNA in primary cancers was significantly associated with earlier biochemical recurrence in a recent data set from Taylor et al.,^13^ with a supportive trend in two additional smaller data sets (Supplementary Fig. 5D–F)^39, 41^.

We next explored whether the decreased AIM1 expression was associated with prostate cancer genetic or epigenetic alterations. Loss of genomic segments on the q-arm of chromosome 6 has been shown to occur frequently in both primary prostate cancer and even more so in metastatic prostate cancer^11^. Mining publically available databases, we identified *AIM1* as a gene located in the core-deletion region on chromosome 6q (Supplementary Fig. 5G). AIM1 copy-number loss was observed in 21–32% of primary cancers, and in 39–43% of metastatic prostate cancers (Fig. 8d)^13, 40, 42–44^. Interestingly, there was a strong direct correlation between *AIM1* mRNA levels and *AIM1* copy-number loss in primary prostate cancers, which could also be confirmed in paired analysis of tumors and associated tumor-adjacent benign tissues (Supplementary Fig. 5H–J)^13^. In these paired analyses, the primary prostate cancers also showed higher *AIM1* mRNA expression than matched benign tissue, particularly in cases without *AIM1* copy-number loss. Taken together, these findings suggest that in primary cancers, AIM1 expression is largely retained but shows dysregulated protein localization, and that in metastatic cancers, this mislocalization is compounded by an increased tendency to lose AIM1 expression and copy number.

Since previous reports suggested that the *AIM1* promoter undergoes CpG island methylation during imprinting in the placenta^45^ and in cancer^46, 47^, we evaluated the CpG methylation status near the *AIM1* transcriptional start site (TSS) (Supplementary Fig. 6A, B). Although we could identify an *AIM1* TSS-proximal CpG island at which methylation was inversely correlated with mRNA expression in prostate cancer cell lines (Supplementary Fig. 6C, D), hypermethylation at this region was not observed in primary tumors (0/20) or metastases (0/24) (Supplementary Fig. 6E). This suggests that copy-number loss and other mechanisms are more important than CpG island hypermethylation in reducing *AIM1* expression in human prostate cancer.

Overall, the observations of AIM1 dysregulation through mislocalization, reduced expression, and genomic loss suggests that initial mislocalization and dissociation of AIM1 away from the actin cytoskeleton can promote formation of invasive carcinoma and high-grade cancers, and that further loss of AIM1, through decreased gene expression and/or genomic deletion, can be associated with development of recurrence and formation of metastases.

## Discussion

Cancer cells must acquire the ability to invade through normal architectural confines in order to establish invasive carcinoma, local extension, and distant metastases. The ability to dynamically remodel the actin cytoskeleton is a key factor in such cancer cell invasion and understanding the molecular players involved in such processes is of critical importance^1–3, 48, 49^. We now identify a novel factor, AIM1, that binds the actin cytoskeleton and acts to suppress cytoskeletal remodeling and invasive phenotypes in prostate epithelial cells. AIM1 dysregulation, through mislocalization and dissociation from the actin cytoskeleton, or AIM1 reduced expression and genomic loss, was common in prostate cancer and was associated with more aggressive, recurrent, or metastatic disease.

The actin cytoskeleton is a dynamic cellular scaffold that can be modeled as a “soft solid” that can undergo plastic flow following the principles of rheology^50, 51^. By measuring the spontaneous nanoscale tracer motions of a cytoskeleton-tethered magnetic beads attached at the cell surface, we were able to understand the extent of active remodeling of the cytoskeleton in cells with and without loss of AIM1. Interestingly, depletion of AIM1 resulted in an increased dynamic remodeling of the actin cytoskeleton; and traction force microscopy revealed that AIM1 loss increased cellular traction forces. These biophysical changes were associated with an increased level of G-actin, as seen by biochemical fractionation and in situ labeling experiments. Importantly, in a number of studies, the metastatic potential of cancer cells has been closely associated with increased cytoskeletal remodeling and concomitant increase in traction stresses^22, 52, 53^. The increased cytoskeleton remodeling observed in AIM1-depleted cells may result in softening of the cortical actin and dissociation of cell–cell contacts. In addition, the increased formation of focal adhesions together with the augmented myosin activity in AIM1-depleted cells provides an explanation for the increase in traction forces ultimately leading to the migration and invasion phenotype documented here. In summary, these findings suggest that AIM1 is involved in broadly suppressing cellular biomechanical and biochemical properties that have previously been associated with increased cell migration and metastatic potential^5, 50, 54–58^. This was further confirmed by our observations that AIM1 loss significantly increased cellular invasiveness and anchorage-independent growth. Our results therefore highlight the role of accurate biophysical measurements as a way to predict phenotypic responses and shed light on the molecular function of AIM1.

While the precise mechanisms by which AIM1 suppresses actin cytoskeletal remodeling and cell invasive phenotypes require further investigation, our data suggest that this occurs through its direct interaction with the actin cytoskeleton. Using biochemical and imaging approaches, we provide evidence that AIM1 forms a complex with the actin cytoskeleton in vitro^18^. Furthermore, a deletion mutant (Δ859) in which all of the βγ-crystallin domains of AIM1 were deleted failed to associate with the actin cytoskeleton and failed to rescue AIM1 loss-driven phenotypes. These data suggest that AIM1 interaction with the actin cytoskeleton through its βγ-crystallin domains is essential for AIM1-mediated suppression of actin cytoskeletal remodeling and invasive phenotypes. βγ-crystallin proteins are the major structural constituents of the eye lens^59^. Interestingly, AIM1 is the only βγ-crystallin domain containing protein seen outside of the vertebrate lens. Given the role of βγ-crystallins as structural proteins in the lens, it is intriguing that our results indicate that AIM1 plays a key role in controlling cellular structure. In vivo co-localization and proximity ligation experiments strongly support our in vitro observations and show that AIM1 interacts with the actin cytoskeleton in normal prostate epithelial cells, where it is likely involved in establishing the stable 3D glandular architecture of the epithelial cells while suppressing invasive properties. Our unbiased interaction screen revealed a number of AIM1-associated proteins that have diverse cellular functions, a finding that will need additional confirmation in future studies using multiple cell lines and additional replicates. Of particular interest is the interaction with filamins A/B. In general, filamins have been shown to influence diverse signaling pathways by functioning as a scaffold for multiple signaling intermediates^60^. The interaction of PARP1 with AIM1 is also of significant interest given the role of PARP1 in nuclear hormone receptor signaling and the recent clinical responses seen with PARP inhibitors in solid tumors^61^. Future studies examining in greater detail the spectrum of AIM1 interactions will provide additional insights into the mechanistic roles of AIM1 apart from actin cytoskeleton modulation.

In human prostate cancers, AIM1 function appears to become dysregulated at multiple levels. First, nearly universally in all of the prostate cancer tissues we examined, AIM1 became dissociated from the actin cytoskeleton and lost the peri-membranous localization seen in normal epithelia, instead showing a more diffuse cytoplasmic localization. Interestingly, this change in AIM1 localization between normal and malignant prostate epithelium closely mimicked the change in AIM1 localization seen in phases of cell invasion during prostate development. This parallelism supports the notion that prostate cancer cells might reawaken an embryonic program to facilitate migration by signaling dissociation of AIM1 from the actin cytoskeleton. In support of this, a greater cytoplasmic relative to membranous localization of AIM1 in prostate cancer tissues was strongly associated with higher histological grade. The precise signaling mechanisms mediating dissociation of AIM1 from the actin cytoskeleton requires further investigation. Second, AIM1 mRNA expression levels were reduced in a fraction of prostate cancer cases, particularly in metastatic disease. Lower AIM1 mRNA expression in primary prostate cancer was associated with biochemical recurrence. Importantly, in two independent xenograft models, we show that RNAi-mediated reduction of AIM1 expression resulted in a dramatically increased amount of micrometastases, supporting the notion that AIM1 could be involved in different stages of metastatic dissemination. Finally, AIM1 loss could become reinforced at the genetic and epigenetic levels. Approximately 30% of primary prostate cancers and >50% of metastatic prostate cancers harbor alterations of the AIM1 gene locus. In addition to copy-number loss, recent studies suggest that AIM1 transcriptional regulation might be in part mediated by CpG island methylation-based silencing^16, 17, 46^. Our data suggest that a subset of prostate cancer cell lines, but not primary or metastatic prostate cancer tissues, showed promoter CpG island hypermethylation of the AIM1 gene associated with its transcriptional silencing. Thus, it appears that initial dysregulation of AIM1 function through mislocalization can become further enforced through reduced AIM1 expression and/or genomic deletion in prostate cancer cases.

While a previous report has suggested that loss of AIM1 could mediate a modest reduction in the viability of VCaP prostate cancer cells^62^, our data show that the major effect of AIM1 loss is a substantial increase in cell migration and invasiveness through increased cytoskeletal remodeling. These observations suggest that AIM1 plays a key role in suppressing migration, invasion, and micrometastatic dissemination phenotypes and thus nominate AIM1 as an important tumor and metastasis suppressor.

## Methods

### Cell culture and transfection

HEK293 cells were obtained from the ATCC (Manassas, VA) and were grown in DMEM medium (Invitrogen, Carlsbad, CA) containing 10% fetal bovine serum. Normal HPV-immortalized prostate epithelial cells (RWPE-1, 957) were obtained from Dr. John Isaacs (Johns Hopkins University, Baltimore, USA) and cultured in Keratinocyte SFM medium containing 0.2 ng/ml epidermal growth factor (EGF) and 25 mg/ml bovine pituitary extract (catalog no. 17005-075, Life Technologies)^63^. VCaP and PC3 cells were obtained from the ATCC (Manassas, VA) and were grown in RPMI medium containing 10% fetal bovine serum. Cell line authenticity and mycoplasma contamination testing was routinely confirmed by PCR-based assays and STR genotyping, respectively, in 6–10-month intervals. Plasmids coding for AIM1 targeting sh-RNAs (RHS3979-99220194, RHS3979-99220202, RHS3979-99220210, and RHS3979-99220274) and non-targeting controls (RHS4080 and RHS4459) were purchased from OpenBiosystems (Lafayette, CO). RWPE-1 cells were transfected with lipofectamine (Life Technologies) and stable clones were selected in 1 µg/ml puromycin (Sigma Aldrich). Separately, RWPE-1 cells were also transduced using GIPZ Lentiviral Particles targeting AIM1 (V3LHS_370583 and V3LHS_370585) and non-silencing controls (RHS4348) (OpenBiosystems). Additional sh-RNA constructs were purchased from Transomics. GFP-positive cells were enriched by fluorescence-activated cell sorting and knockdown efficiency was monitored by quantitative real-time PCR and western blotting. Human *AIM1* (NM_001624) and *ACTB* (NM_001101) expression clones were obtained from GeneCopoeia (Rockville, MD) and were subcloned into pcDNA3.2/capTEV-NT/V5-DEST vectors using the Gateway cloning system (Life Technologies). Deletion constructs in pEZM15-AIM1 and pcDNA3.2/capTEV-AIM1 vectors were generated by digesting with NotI and AgeI for Δ1287 and NotI and PshA1 for Δ859 (New England Biolabs, Ipswich, MA).

### Antibodies

Mouse monoclonal anti-beta actin (1:500, clone 8H10D10) and rabbit polyclonal antibodies (1:300, 13E5) were obtained from Cell Signaling (Beverly, MA). G-actin-specific mouse monoclonal antibodies (1C7) were obtained from the Antibody Facility TU Braunschweig (1:50, Braunschweig, Germany)^21^. Antibodies specific to paxillin (1:200, abcam, ab32084), phospho-paxillin (Tyr118) (1:100, Cell Signaling, #2541), phospho-myosin light chain 2 (1:200, Thr18/Ser19) (Cell Signaling, #3674), phospho-FAK (Tyr576/577) (1:100, Cell Signaling, #3281), phospho-FAK (Tyr925) (1:100, Cell Signaling, #3284) were used for immunofluorescence microscopy and western blot analyses. For the detection of phospho-paxillin and phospho-FAK, tyramide signal amplification using the TSA kit (Perkin Elmer) was performed. Custom-made rabbit polyclonal antibodies against an N-terminal peptide sequence (JH6528, aa 395–417) of AIM1 (LIPVKDHKLLEKEDSEAADSKS) were raised in rabbits and affinity purified by Covance (Gaithersburg, MD) and used at 1:100 and 1:300 for immunofluorescence microscopy and western blot, respectively. Additional custom-made rabbit monoclonal antibodies were raised against a highly conserved N-terminal peptide sequence of AIM1 (CKLNLAKKAKEMEQPEKK). Hybridomas were generated by Abcam (Burlingame, CA) and propagated using previously established protocols. Images of uncropped western blot gels can be found in Supplementary Fig. 7.

### Unbiased proteomic interaction screen

Human *AIM1* (NM_001624) and *ACTB* (NM_001101) expression clones were obtained from GeneCopoeia (Rockville, MD) and were subcloned into pcDNA3.2/capTEV-NT/V5-DEST vectors using the Gateway cloning system (Invitrogen). RWPE-1 cells were transfected with pcDNA3.2/capTEV-ACTB vectors using Lipofectamin 2000. Forty-eight hours after transfection cells were lysed in CoIP lysis buffer (50 mM HEPES, pH 7.5, 1 mM EGTA, 2 mM EDTA, 12.5 mM β-glycerophosphate, 3.2 mM MgCl_2_, 10% glycerol, 1% Triton X-100, and 1× Complete EDTA-Free Protease Inhibitor cocktail (Roche, Basel, Switzerland)). Complexes associated with endogenously biotinylated actin or AIM1 proteins were first immobilized on Streptavidin beads (Life Technologies), washed four times in Elution Buffer and eluted by incubation with recombinant AcTEV protease (Life Technologies). Eluates containing high-molecular-weight AIM1-associated protein complexes were subjected to 60 kDa size selection and recovered proteins were identified by liquid chromatography tandem mass spectrometry (LC-MS/MS). In brief, to each sample of protein in solution (20 μl) 1 μl of 1 M ammonium bicarbonate was added. The proteins were then reduced with 2 μl TCEP for 60 min at 60 C, alkylated with 1 μl MMTS at room temperature for 20 min, and digested with trypsin (Promega) as previously described at 37 °C overnight^64^. Digested peptides were acidified and dried by speedvac. Protein identification by LC-MS/MS analysis of peptides was performed using an LTQ ion trap MS (Thermo Fisher Scientific) interfaced with a 2D nanoLC system (Eksigent, Dublin, CA). Peptides were loaded on a 75 μm × 2.5 cm trap packed with YMC*GEL ODS-A 12 nm S-10 μm C18 material and then fractionated by reverse-phase HPLC. Peptide sequences were identified using Mascot (www.matrixscience.com) software using the RefSeq_40_ complete_20100416 database. Mascot search results were processed in Scaffold (www.proteomesoftware.com) to validate protein and peptide identifications. Proteins with identification of at least two peptides at 95% or greater confidence were considered “hits”. Supplementary Table 1 shows the number of recovered peptide tags for both AIM1-bait and control-bait pulldown reactions.

### Co-immunoprecipitation

Co-immunoprecipitation experiments were performed from total cell lysates prepared in 50 mM HEPES, pH 7.5, 1 mM EGTA, 2 mM EDTA, 12.5 mM β-glycerophosphate, 3.2 mM MgCl_2_, 10% glycerol, 1% Triton X-100, supplemented with 1× Complete protease inhibitor cocktail (Sigma Aldrich). Lysates were incubated with mouse-monoclonal anti-β Actin antibodies (8H10D10, Cell Signaling, Beverly, MA), AIM1-specific antibodies or control mouse gamma globulin over night at 4 °C and immune complexes were collected by adding Protein G Plus agarose beads (Santa Cruz Biotechnology) for 4 h followed by centrifugation. Immobilized immune complexes were washed, eluted by boiling in 1% SDS, and separated by SDS-PAGE. Immunoblots were probed with indicated antibodies.

### Matrigel invasion assay

Reduced Growth Factor Basement Membrane Matrix (Geltrex, Invitrogen) was diluted to a final concentration of 5 mg/ml in cold serum-free medium and 100 μl of matrigel suspension was layered in the top insert of a 8 μm pore size transwell (Costar Corning). Twenty-thousand cells were resuspended in 100 μl of serum-free medium and gently layered on top of the matrigel. The top part of the transwell chamber was filled with serum-free medium, the bottom well was filled with fully supplemented medium containing EGF, pituitary extract and 5 µg/ml fibronectin (Sigma-Aldrich). For collagen matrix experiments, the collagen I kit from the Trevigen (3457-024-K) was used. Laminin (Sigma, L2020) was applied at a final concentration is 20 μg/ml. Forty-eight hours after cell seeding, the matrigel was removed and invaded cells were fixed with 4% formaldehyde stained with either DAPI or hematoxylin and counted at ×20 magnification. Spheroid invasion assays were carried out using the 3D spheroid BME cell invasion assay (Trevigen catalog no. 3500-096-K). In brief, cells were trypsinized, pelleted, and resuspended in 50 μl of complete media + 1X Spheroid Formation ECM, and then cultured for 3 days at 37 ^o^C/5% CO_2_, at a density of 2000 cells per well. After 3 days, spheroids were transferred into the invasion matrix and imaged after incubating for 6 days.

### Soft agar assay

Cells were layered in supplemented medium containing 0.6% agarose (Life Technologies) and incubated for 14 days and media was renewed every 3 days. Resulting colonies were stained with 0.005% Crystal Violet (Sigma) for 2 h and counted.

### Transmission electron microscopy

Cells were fixed in 2.5% glutaraldehyde, 3 mM MgCl_2_ in 0.1 M sodium cacodylate buffer, pH 7.2 for 1 h at room temperature. After buffer rinse, samples were post-fixed in 1% osmium tetroxide in buffer on ice in the dark. Following a DH_2_O rinse, samples were en bloc stained with 2% aqueous uranyl acetate (0.22 µm filtered), dehydrated in a graded series of ethanol and embedded in Eponate 12 (Ted Pella) resin. Samples were polymerized at 37 °C for 2–3 days followed by 60 °C overnight. Thin sections, 60–90 nm, were cut with a diamond knife on the Reichert-Jung Ultracut E ultramicrotome and transferred to a copper slot grids. Grids were stained with 2% uranyl acetate in 50% methanol followed by lead citrate and observed with a Philips CM120 TEM at 80 kV. Images were captured with an AMT CCD (8 megapixel camera—side mount AMT XR80—high-resolution high-speed camera).

### Biochemical G-Actin and F-Actin fractionation

Biochemical fractionation experiments were carried out using the G-actin/F-actin in vivo assay kit from Cytoskeleton, Inc. (Denver, CO) following the manufacturer’s protocols.

### Spontaneous nanoscale tracer motions

To evaluate the remodeling of the cytoskeleton we used spontaneous nanoscale tracer motions (SNTM). RGD-coated microbeads were anchored to the cytoskeleton through cell surface via integrin receptors. Spontaneous nanoscale movements of individual beads (~4.5 µm in diameter) bound on adherent cells (~50–100 beads per field of view) were recorded. The trajectories of bead motions in two dimensions were used to compute the mean square displacement of all beads as function of time [MSD(*t*)] (nm^2^), as previously described^23^. Herein, we analyzed MSD data for times >10 s and up to 300 s. In addition, diffusion coefficient D* and the exponent *α* of the bead motion were estimated from a least-square fit of a power-law to the ensemble average of MSD data vs. time^23^.

### Fourier transform traction microscopy

The distribution of traction fields arising at the interface between each adherent cell and its substrate were evaluated using FTTM. In brief, cells were plated sparsely on gel blocks, and allowed to adhere and stabilize for 24 h. For each adherent cell, images of fluorescent microbeads (0.2 µm in diameter, Molecular Probes, Eugene, OR) embedded near the gel apical surface were taken at different times; the fluorescent image of the same region of the gel after cell detachment with trypsin was used as the reference (traction-free) image. The displacement field between a pair of images was then obtained by identifying the coordinates of the peak of the cross-correlation function^26^. From the displacement field and known elastic properties of the gel (Young’s modulus of 1300 Pa with a Poisson’ ratio of 0.48) the traction field was computed using both constrained and unconstrained FTT cytometry^26^. The computed traction field was used to obtain net contractile moment, which is a scalar measure of the cell’s contractile strength. Net contractile moment is expressed in units of pico-Newton meters (pNm).

### Xenograft experiments

All the animal experiments were performed according to protocols approved by the Animal Care and Use Committee at Johns Hopkins University. Athymic male nude mice (nu/nu, 8 weeks old) were obtained from Envigo (Huntingdon, UK) and maintained in a sterile environment. Cell lines stably expressing shRNAs targeting AIM1 or control non-targeting vectors were generated as described above. RWPE-1, PC3 and VCaP cells (1 × 10^6^ cells in 80% Matrigel, 20% PBS) were then injected in the mouse flank. A 1:1 randomization (sh-AIM vs. sh-control) was used for all experiments. Since no prior experiments were available to estimate anticipated effect size and because we considered large effect sizes to be meaningful, five animals per group were used (giving 76.6% power to see a 1.5 standard deviation difference in the mean assuming equal standard deviation in each group). Caliper tumor size measurements were performed once a week and tumor volumes were calculated. At the time of necropsy (4 weeks after inoculation for PC3 xenografts, 6 weeks for VCaP xenografts), tumor tissues from the flank, as well as lung, liver, and spleen were harvested and fixed in 10% buffered formalin or snap-frozen in liquid nitrogen. No animals were excluded from the analysis. Personnel involved in animal experiments were blinded to the cell phenotype (knockdown vs. control) during engraftment and downstream analyses.

### PCR-based measurement of micrometastatic burden

DNA extractions from frozen tissue samples were performed using QIAshredder columns for tissue homogenization and DNeasy extraction kits for DNA purification (Qiagen, Hilden, Germany) following manufacturer’s instructions. Extracted DNAs were quantitated using Qubit fluorometric quantification kits (Invitrogen) and quantitative real-time PCR. The abundance of *Alu* DNA elements in murine tissue samples was quantitated using a Taqman-based real-time PCR assay using 1× Master Mix for PCR (Biorad), 200 nM of forward and reverse primers (hALU_F: GTCAGGAGATCGAGACCATCCT, hALU_R: AGTGGCGCAATCTCGGC) and 300 nM of probe (hALU_PROBE: 6-FAM-AGCTACTCGGGAGGCTGAGGCAGGA-TAMRA) under the following conditions: 95 °C for 5 min, 40 cycles of 95 °C for 20 s, 60 °C for 40 s. Note that primer and probe sequences used here were described previously^34^. DNA samples with known cell equivalent amounts for VCaP and PC3 cells were analyzed as a reference.

### Wound healing assay

For wound healing assays, cells were plated into six-well dishes. When a confluency of 80–90% was reached cells were washed with serum-free medium two times for 1 h. A 1–2 mm wound was then scratched into the cell monolayer and fresh fully supplemented medium was added. Cells were then imaged on a Nikon Eclipse TE-2000E live cell microscope equipped with NIS-Elements AR 3.10 software at ×20 magnification for 24 h. Images were taken every 10 min. Representative quicktime time-lapse movies are provided in the Supplementary Materials.

### 3D cultures

3D culture experiments were performed by seeding cells onto matrigel (BD Biosciences) supplemented with 1.6 μg/ml collagen I (BD BioScience). Cultures were grown for 6–8 days in medium containing 2% matrigel, 2% FBS and 5 ng/ml EGF^32, 65^. Cells were then fixed with 4% formaldehyde and stained directly in situ with rhodamine phalloidin (Life Technologies) and DAPI. Slides were then visualized using a Nikon Eclipse TE-2000E confocal microscope equipped with the EZ-C1 3.90 software.

### Live cell micromechanical methods

SNTMs of beads bound to the surface of RWPE-1 cells expressing either control sh-RNAs or sh-AIM1 cells were recorded longitudinally from 10 to 300 s and MSD of all beads were calculated as a function of time^23^. For FTTM, cells were plated on elastic polyacrylamide 24 h prior to imaging^66^. Embedded fluorescent microbeads (Molecular Probes, Eugene, OR) were imaged and compared to images after cell detachment by trypsinization (traction-free)^26^. Based on the displacement of the fluorescent microbeads and the known elastic properties of the matrix, a traction field was calculated and used to obtain the net contractile moment, expressed in picoNewton-meters (pNm), which is a scalar measure of the cell’s contractile strength^26^.

### Immunohistochemistry

COS7 cells grown on glass coverslides were transfected with Lipofectamine 2000 (Life Technologies). 24 h after transfection, cells were fixed with 4% para-formaldehyde and permeablized with 0.125% Triton X-100 in PBS. Permeabilized cells were then blocked in 1% BSA containing PBS for 1 h. Cells were then further incubated with rhodamine phalloidin (Life Technologies) at 1:400 dilution for 1 h at room temperature, counterstained with DAPI and mounted with Prolong Gold (Life Technologies). Immunohistochemical detection of AIM1 in FFPE was first optimized on HEK293 cells overexpressing AIM1 and RWPE-1 cells depleted of AIM1 by shRNA (Supplementary Fig. 4) that were fixed in formalin and embedded in paraffin. Staining conditions included de-paraffinization in Xylene and rehydration and steaming in 10 mM sodium citrate (pH = 6) for 30 min. The primary antibody (anti-AIM1, JH6528) was applied at 1:4000 for 1 h. Immuno-complexes were then visualized with the EnVision + kit (DakoCytomation, Carpinteria, CA) with DAB as the chromogen. For fluorescence microscopy we used primary anti-AIM1 rabbit polyclonal antibodies (JH6528) and anti-beta actin antibodies (8H10D10, Cell Signaling) at 1:500 and 1:1500 for 1 h, respectively. Alexa-Fluor-488-labeled anti-mouse and Alexa Fluor-568 anti-rabbit (Life Technologies) secondary antibodies were applied at 1:100 for 30 min. Slides were counterstained with DAPI and mounted with Prolong Gold (Life Technologies).

### Tissue microarrays

This study was approved by the Johns Hopkins Medicine Institutional Review Board. Consent for correlative tissue studies was obtained. In total, 508 tissue microarray (TMA) spots representing 81 normal prostate tissues, 87 primary carcinoma, and 52 lymph-node metastases from 104 patients were analyzed (comprising 51 cases with matched normal prostate/prostate cancer/lymph-node metastasis tissue spots; 13 cases with matched normal prostate/prostate cancer tissue spots; 1 case with matched prostate cancer/lymph-node metastasis tissue spots; 22 cases with only prostate cancer tissue spots; and 17 cases with only normal prostate tissue spots). All cases were read and evaluated by an experienced uro-pathologist (AC).

### Proximity ligation assay

Proximity ligation was performed using the DUOLinkTM kit following manufacturer’s protocols (OLINK, Uppsala, Sweden)^35^. Slides were deparaffinized and steamed in 10 mM sodium citrate (pH 6) for 30 min. After blocking (DUOLinkTM kit), AIM1 (JH6528) ([1:100]and β-actin ([1:10], Cell Signaling) antibodies were incubated for 1 h at room temperature. PLA reactions were then carried out according to manufacturer’s instructions.

### Fluorescence and bright field imaging

Images were captured using a Nikon E400 fluorescence/bright field microscope equipped with a Nikon DXM1200 camera (Nikon Instruments, Melville, NY) and the SPOT Advanced digital imaging software (Diagnostic Instruments, Inc., Sterling Heights, MI). Confocal images were obtained using a Zeiss AxioObserver with LSM700 confocal module (Carl Zeiss Microscopy GmbH, Jena, Germany). Signal quantifications were performed using ImageJ and the Fiji image analysis package^67, 68^.

### Statistical analyses

All graphs and statistical analyses for in vitro and in vivo experiments were generated using Prism 6.0 (GraphPad Softward, Inc., La Jolla, CA) using *t*-test statistics. For analysis of immunohistochemical expression data, since scores were non-normally distributed, non-parametric tests were used to compare their differences depending on the examined tissue. Comparisons were made for membranous and cytoplasmic AIM1 expression. Scores between benign prostate, prostate cancer, and lymph-node metastasis were compared using the Kruskal–Wallis test, adjusting for tied values. Pairwise comparisons were done using the Wilcoxon rank-sum (Mann–Whitney *U*) test. A two-tailed *P* < 0.05 was considered statistically significant, except for post hoc comparisons. For the latter, the significance threshold was adjusted using Šidák’s correction, and set to *P* < 0.017. Data were analyzed using Stata/SE 11.2 for Windows (StataCorp LP, College Station, TX). Gene set enrichment analysis was carried out using the DAVID functional annotation tool^69^.

### Data availability

All reagents and data are available upon request.

## Electronic supplementary material

Supplementary Information

Supplementary Movie 1

Supplementary Movie 2
