# Supplementary material for: AIM1 is an actin-binding protein that suppresses cell migration and micrometastatic dissemination
Source: Nat Commun. 2017 Jul 26;8:142. doi: 10.1038/s41467-017-00084-8 (PMC5529512; doi:10.1038/s41467-017-00084-8)
Supplement: Supplementary file 1 — Supplementary Information [file 41467_2017_84_MOESM1_ESM.pdf]

Title: Supplementary Information

Description: Supplementary Figures and Supplementary Tables

Title: Supplementary Movie 1

Description: Wound closure experiment showing delayed closure of “wound” scratched into a monolayer of RWPE1 cells expressing sh-control constructs.

Title: Supplementary Movie 2

Description: Wound closure experiment showing rapid closure of “wound” scratched into a monolayer of RWPE1 cells expressing sh-AIM1 constructs.

A

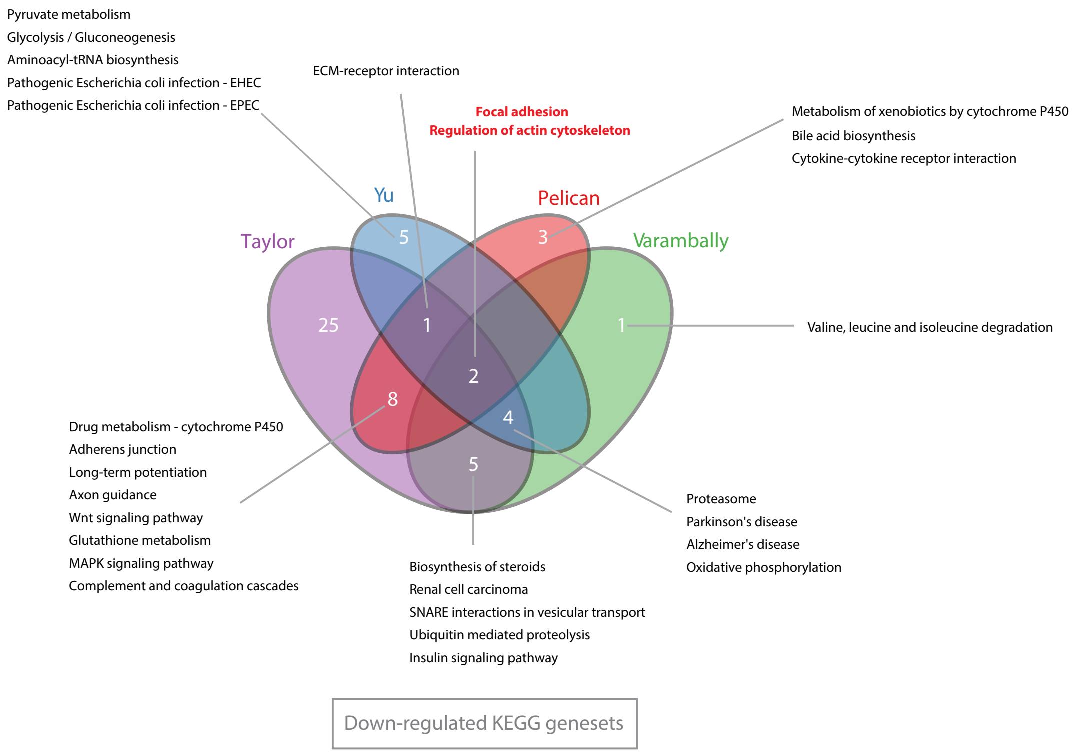

B

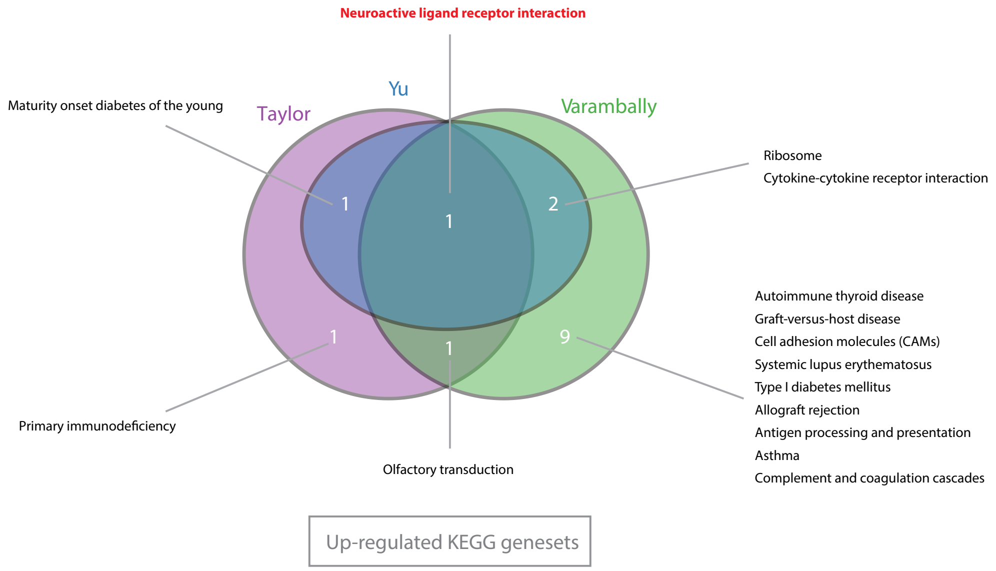

Supplementary Figure 1. Geneset enrichment analyses of multiple gene expression datasets. Geneset enrichment analysis (GSEA) has provided a valuable tool to understand biologically relevant processes that are coregulated on a transcriptional level in different disease states (1). To understand the genesets that are altered between normal prostate epithelium, prostate cancer and prostate cancer metastases we performed GSEA on four large, previously published expression datasets (2-5). When we intersected the enriched KEGG categories from all 4 datasets we observed that only two KEGG genesets ("Focal adhesion" and "Regulation of actin cytoskeleton", $q < 0.05$) were concordantly down regulated in all datasets. This observation suggests that transcriptional dysregulation of a large set of genes involved in the regulation of actin cytoskeletal dynamics could play a major role in prostate cancer progression. It is worth noting that similar changes in geneset enrichment were also observed between the normal prostatic epithelial cells (PrEC) and two well characterized prostate cancer cell lines (LNCaP and LAPC4, data not shown). (A, B) Venn diagram shows number and corresponding up or downregulated KEGG genesets comparing normal prostate to primary prostate cancer (Yu et al., Taylor et al., Varambally et al.) (2-4) and normal prostate and distant prostate metastases (Aryee et al.) (5). Only KEGG genesets that meet the FDR significance cutoff level of $q \leq 0.05$ are listed.

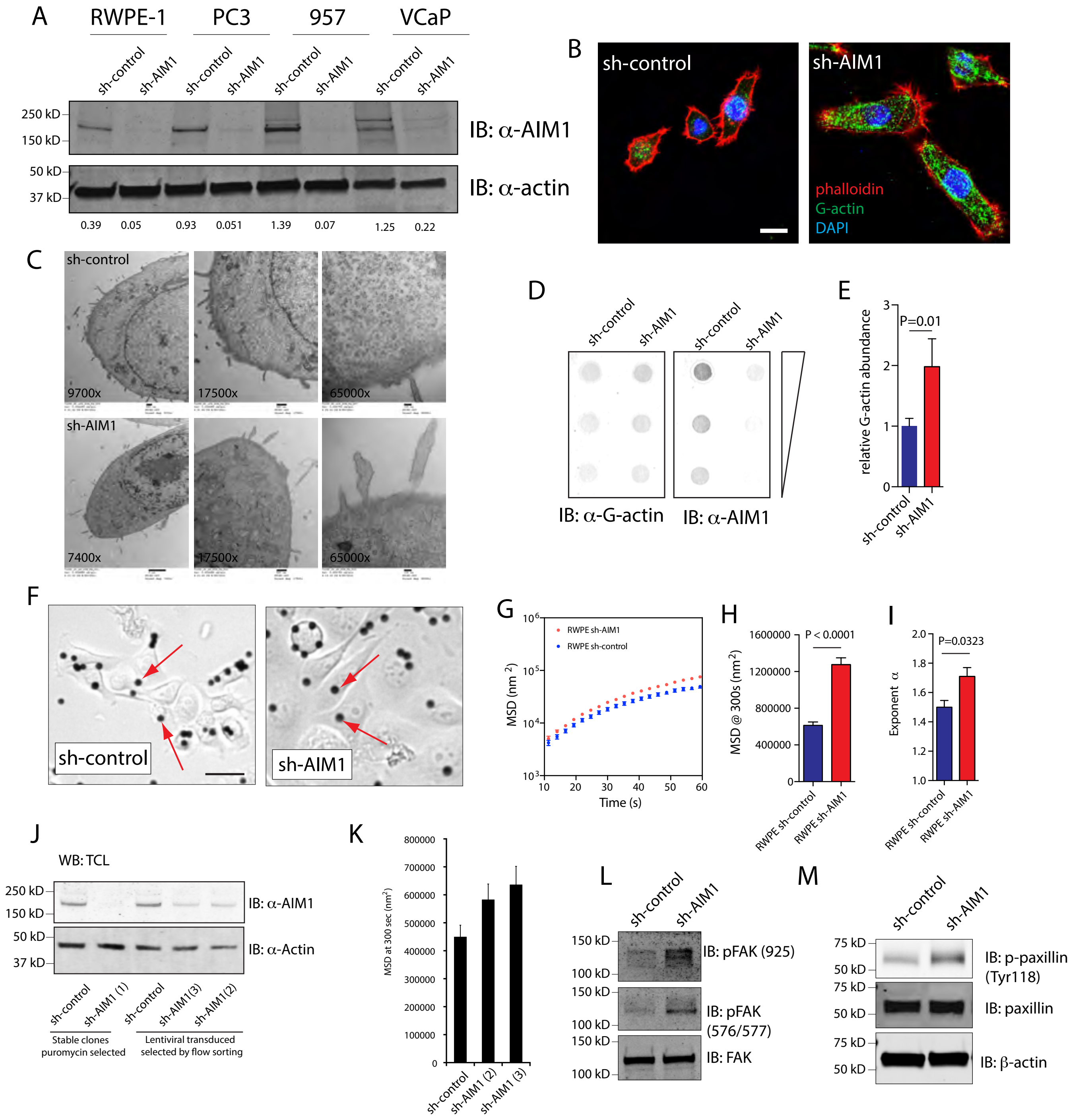

Supplementary Figure 2. AIM1 depletion in RWPE-1 cells results in increased cytoskeletal remodeling and in changes in the G/F actin ratio.

(A) Western blot analysis of RWPE-1, PC3, 957 and VCaP cells overexpressing sh-control and sh-AIM1 constructs. Note the robust depletion of AIM1 protein levels (average reduction of 72 to 92 %) in all cell lines expressing sh-AIM1 constructs . (B) Additonal representative micrographs (similar to micrographs shown in **Figure 3**) demonstrating increased formation of lamellipodia in sh-AIM1 cells. (C) Electron microscopic micrographs of sh-AIM1 and sh-control cells. (D) To determine the abundance of G-actin in RWPE-1 cells stably expressing sh-RNAs targeting AIM1 or a non-targeting sh-RNA, equal numbers of cells were lysed. In a 3 step dilution series, equal amounts of protein from sh-AIM1 and sh-control lysates were spotted onto a nitrocellulose membrane by dot-blotting. After blocking, membranes were incubated with a G-actin specific antibody (clone 1C7, Schoenenberger et al.) (6) or AIM1 specific antibodies; signal intensities of immunoreactive spots were quantitated using an Odyssey fluorescence scanner. (E) Bargraph shows relative abundance of G-actin specific signal in sh-control and sh-AIM1 cells as mean +/- SD of 3 replicates. Note that sh-AIM1 cells show higher abundance of G-actin. This finding is consistent with results presented in **Figure 3** showing an increased G/F actin ratio based on classical biochemical fractionation. (F) Representative micrographs showing RGD-coated magnetic beads (red arrows) coupled to sh-AIM1 and sh-control RWPE-1 cells illustrating the experimental setup used for spontaneous nanoscale tracer motion (SNTM) analyses shown in **Figure 3E,F**. (G) The mean squared displacement (MSD) of microbeads in shAIM1 cells began to increase compared to that in sh-control cells at time points as early as 20 seconds, and continued to show greater increases throughout the experiment. (H) At 300 seconds, the MSD in sh-AIM1 cells was ~2-fold that in sh-control cells (P<0.0001). (I) The supperdiffusive coefficient α, representing the exponent in the power law function fitting the MSD as a function of time, is significantly higher in the sh-AIM1 compared to sh-control RWPE cells. (J) Western blot analysis confirms robust AIM1 protein depletion in stably selected sh-AIM1 (sh-AIM1(1)) expressing cells and in RWPE-1 cells transduced with 2 independent sh-RNA encoding lentiviruses targeting AIM1 (sh-AIM1(2), sh-AIM1(3)). Note that despite significant reduction of AIM1 protein levels with all constructs, the knock-down efficiency of sh-AIM1(2) and sh-AIM1(3) cells is lower than the AIM1 knock down efficiency observed in sh-AIM1(1) cells. (K) SNTM analysis in sh-AIM1(2) and sh-AIM1(3) show increased spontaneous bead motion as compared to sh-control cells validating the findings shown in **Figure 4** with two additional independent sh-RNAs targeting AIM1. (L,M) AIM1 knock down increased FAK phosphorylation (L), and paxillin phosphorylation (M) in sh-AIM1 cells. Scale bar indicates 50 um. All P-values are derived from t-test statistics. Error bars show mean +/- s.d. for 3-4 replicates.

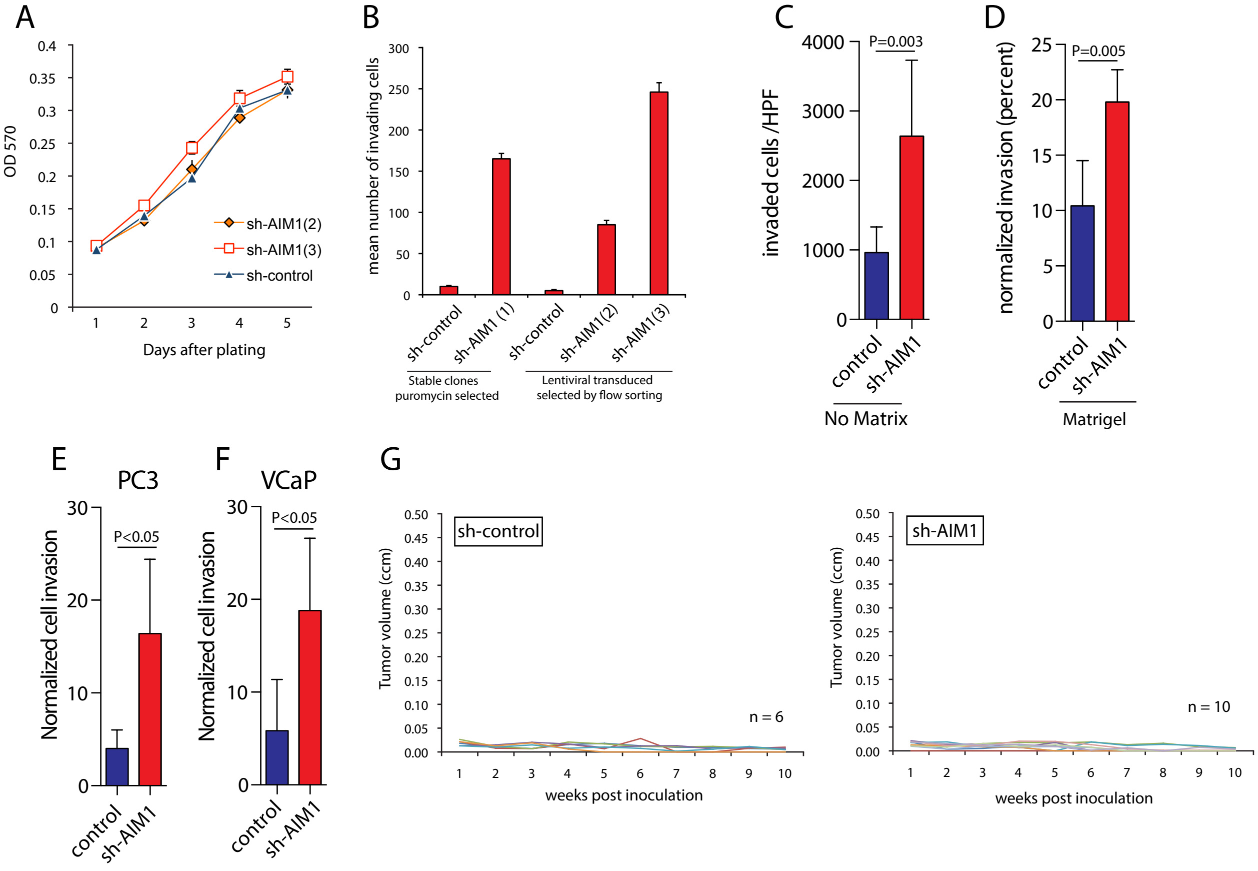

Supplementary Figure 3. AIM1 depletion in RWPE-1 cells results in increased invasion but is not sufficient to enhance cell proliferation or promote xenograft tumor growth.
(A) RWPE-1 cells expressing lentivirus-encoded AIM1 targeting sh-RNAs do not exhibit increased cell proliferation. 1250 cells/well were seeded in 96 well plates and cell abundance was measured by NeutralRed staining every day for 5 days. (B) Classical Boyden chamber experiments reveal increased invasion through matrigel after AIM1 knockdown with 3 independent AIM1 targeting sh-RNAs (for western blot analysis of knockdown efficiency see **Figure 3 and Supplementary Figure 2**). (C) Migration of RWPE-1 sh-AIM1 and sh-control cells through Boyden chambers without any matrix demonstrate increased cell motility of sh-AIM1 cells. (D) Migration-normalized invasion of RWPE-1 sh-AIM1 and sh-control cells through matrigel (see **Figure 5D**). For this graph the number of cells present on the bottom part of Boyden chambers containing matrigel was normalized to the number of cell present on the bottom part of Boyden chambers without matrix. Migration-normalized cell invasion is show as percentage of total cell invasion without matrigel. (E, F) Increased normalized invasion was also observed for PC3 (E) and VCaP (F) cells expressing sh-AIM1 constructs compared to sh-control expressing cells. (B-F) Bar graphs show mean +/- SD of 3 replicates. (G) Knock down of AIM1 in non-malignant prostate epithelial cells (RWPE-1) does not confer tumorigenicity in nude mice. One million RWPE-1 sh-AIM1(1) (n = 10) and sh-control (n = 6) cells were engrafted into the flank of nude mice. Tumor volumes were measured every 7 days for 10 weeks. No tumor growth was noted in any mouse, suggesting that AIM1 depletion alone does not result in full transformation and does not render RWPE-1 cells tumorigenic. All P-values derived from t-test statistics.

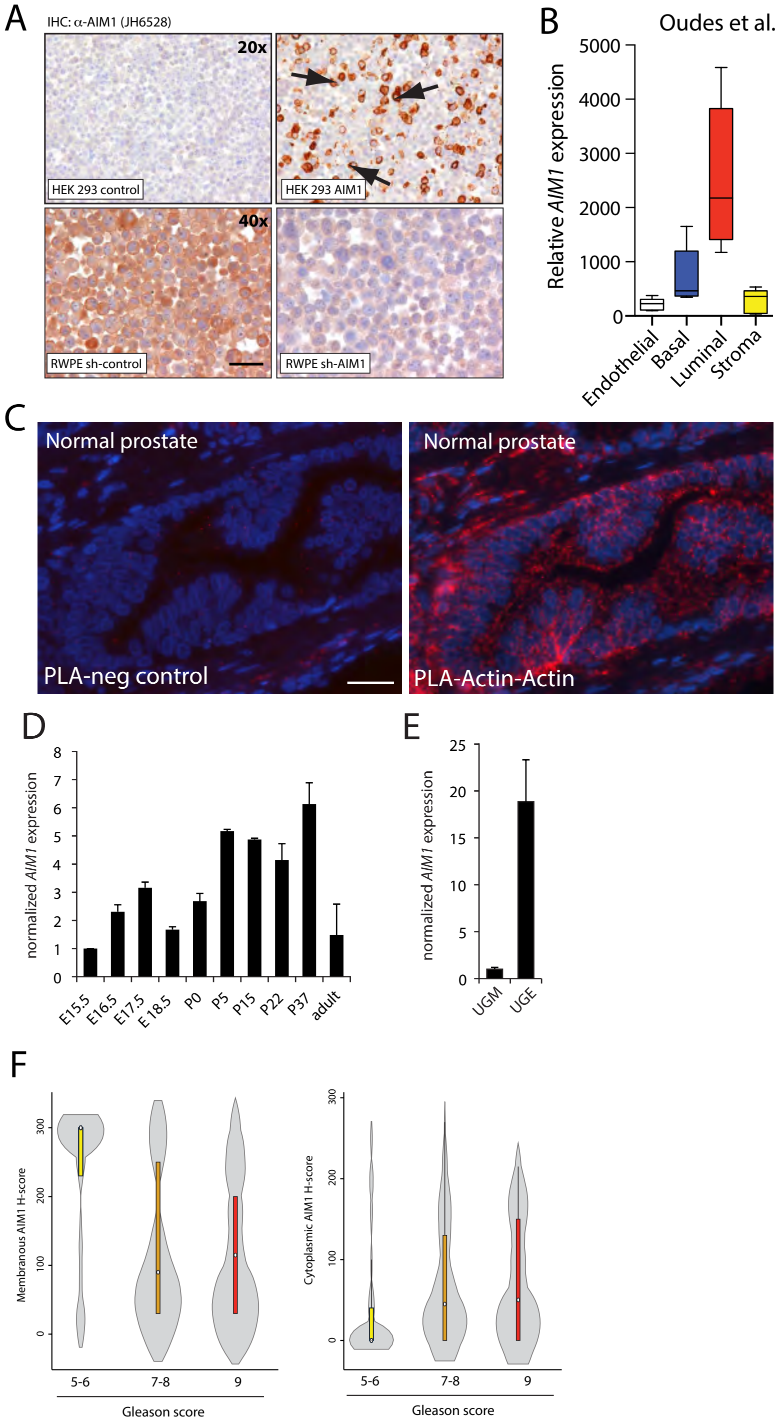

Supplementary Figure 4. Immunohistochemistry and proximity ligation assay controls, AIM1 mRNA expression in mouse development, and AIM1 IHC in clinical specimens stratified by Gleason Score.

(A) Validation of AIM1-specific polyclonal antibodies for immunohistochemistry. To determine the specificity of the custom made AIM1 specific antibody (JH6528), high density FFPE control cell blocks were made using RWPE-1 (high basal AIM1 expression) and RWPE-1 sh-AIM1 cells, and HEK293 (low basal AIM1 expression) and HEK293 cells overexpressing full length AIM1. Note that AIM1 knockdown in RWPE-1 cells results in a profound decrease in immunoreactivity. Conversely, overexpression of AIM1 in HEK293 cells shows strong immunostaining demonstrating the specificity of the antibody. (B) *In silico* analysis of *AIM1* mRNA expression in different cell types of the normal prostate confirms the predominantly luminal expression pattern of AIM1 (for dataset information see Oudes et al.) (7). (C) Specificity controls for proximity ligation assay (PLA). Using two actin specific antibodies (one mouse monoclonal and one rabbit monoclonal), a strong positive signal was generated (PLA Actin-Actin). To test the specificity of the PLA assay, one of the antibodies was omitted, which resulted in a complete absence of PLA specific signals (PLA negative control). (D) Urogenital sinus from mouse embryos (ranging from day 15.5 to 18.5), post-partum (P) days 0 – 37 and adult prostate were analyzed for AIM1 expression using quantitative RT-PCR (n=3 for each timepoint). (E) Aim1 mRNA expression by quantitative RT-PCR in UGM and UGE shows predominant epithelial expression of Aim1 in murine developing prostates (17.5d post-conception, n = 3 animals each). (F) Extent of AIM1 mislocalization is associated with Gleason grade. TMAs containing low- (Gleason score ≤ 6), intermediate- (Gleason score 7) and high-grade (Gleason score 8,9) lesions were stained with AIM1 specific antibodies and immunoreactivity was scored separately for membranous and cytoplasmic localization. Violin-plots show distribution of semi-quantitative H-scores for both compartments. Note that high-grade lesions showed a greater loss of distinct membranous staining and an increased cytoplasmic staining patter. Scale bars indicate 50 um.

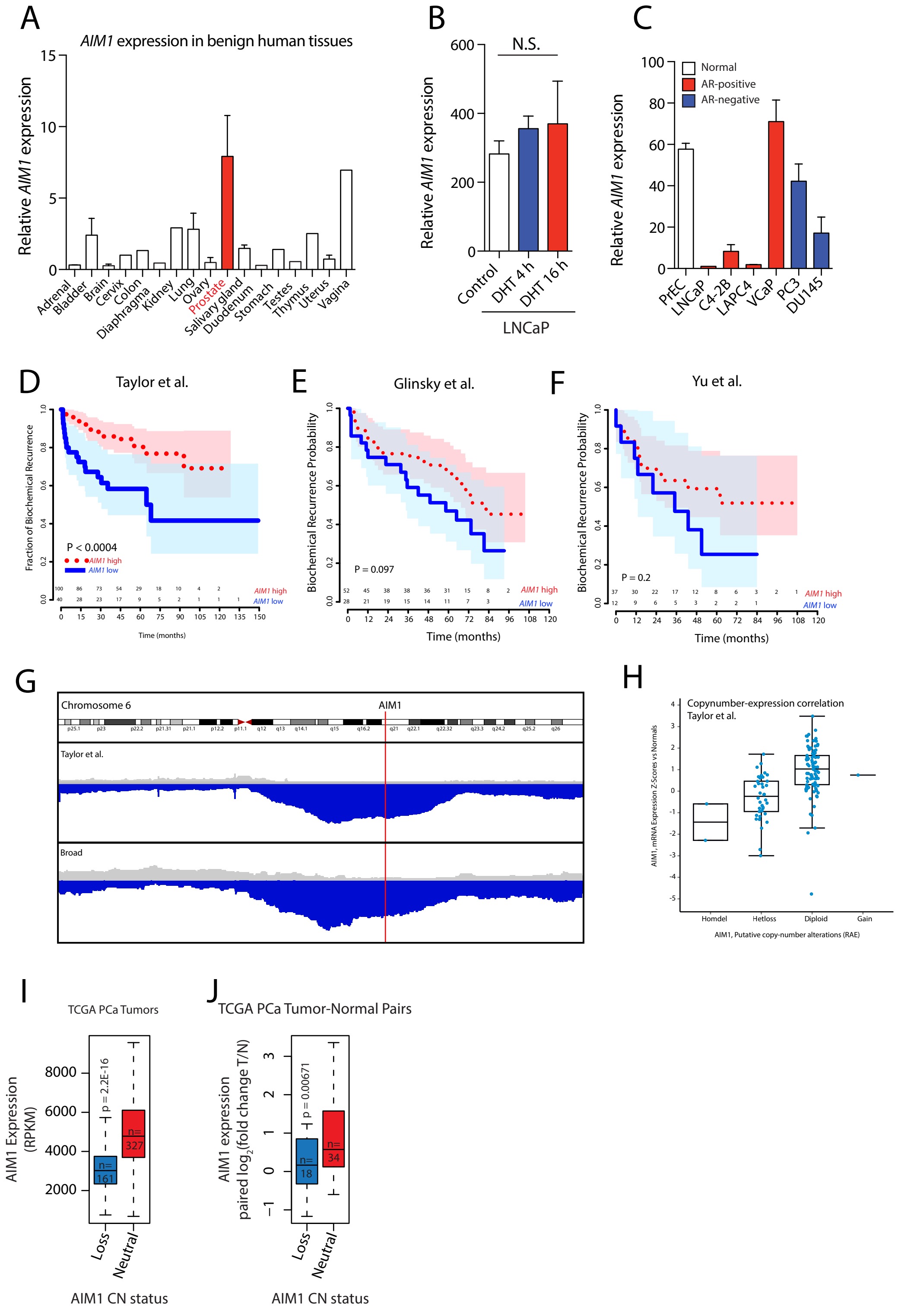

Supplementary Figure 5. Association of AIM1 expression levels with biochemical recurrence after radical prostatectomy and with copy number loss. (A) *In silico* analyses revealed that *AIM1* mRNA expression is high in normal prostate tissue compared to other normal tissue types (8). (B) *AIM1* expression is not significantly regulated by androgen signaling in LNCaP cells. (C) Various prostate cancer cell lines express different levels of AIM1. Note that there is no systematic difference in *AIM1* expression levels between AR-positive and AR-negative prostate cancer cell lines. (D) Low relative mRNA expression of *AIM1* in primary cancers was statistically significantly associated with biochemical recurrence in the Taylor et al dataset (2,9). (E,F) Low relative *AIM1* mRNA expression shows a trend for association with earlier biochemical recurrence in two smaller datasets (Glinsky et al. and Yu et al.) (3,10). (G) AIM1 is part of a core region of frequent genomic deletion in chromosome 6q, as shown in two large prostate cancer copy number datasets (2,11). (H) Homozygous and heterozygous AIM1 loss are strongly associated with reduced AIM1 expression in the Taylor et al. dataset (2,9). (I) *In silico* analyses of AIM1 mRNA expression in the TCGA cohort stratified by copy-number status (12). Note that cases with copy-number loss show significantly lower AIM1 expression. (J) Paired tumor-normal expression analysis in the cases of the TCGA cohort for which matched tumor and adjacent benign expression data was available confirms the association of reduced AIM1 expression with copy number loss. Note the increased AIM1 expression in primary prostate carcinoma compared to adjacent benign prostatic tissue particularly in the copy number neutral cancers. Error bars show mean +/- s.d. for 3-4 replicates.

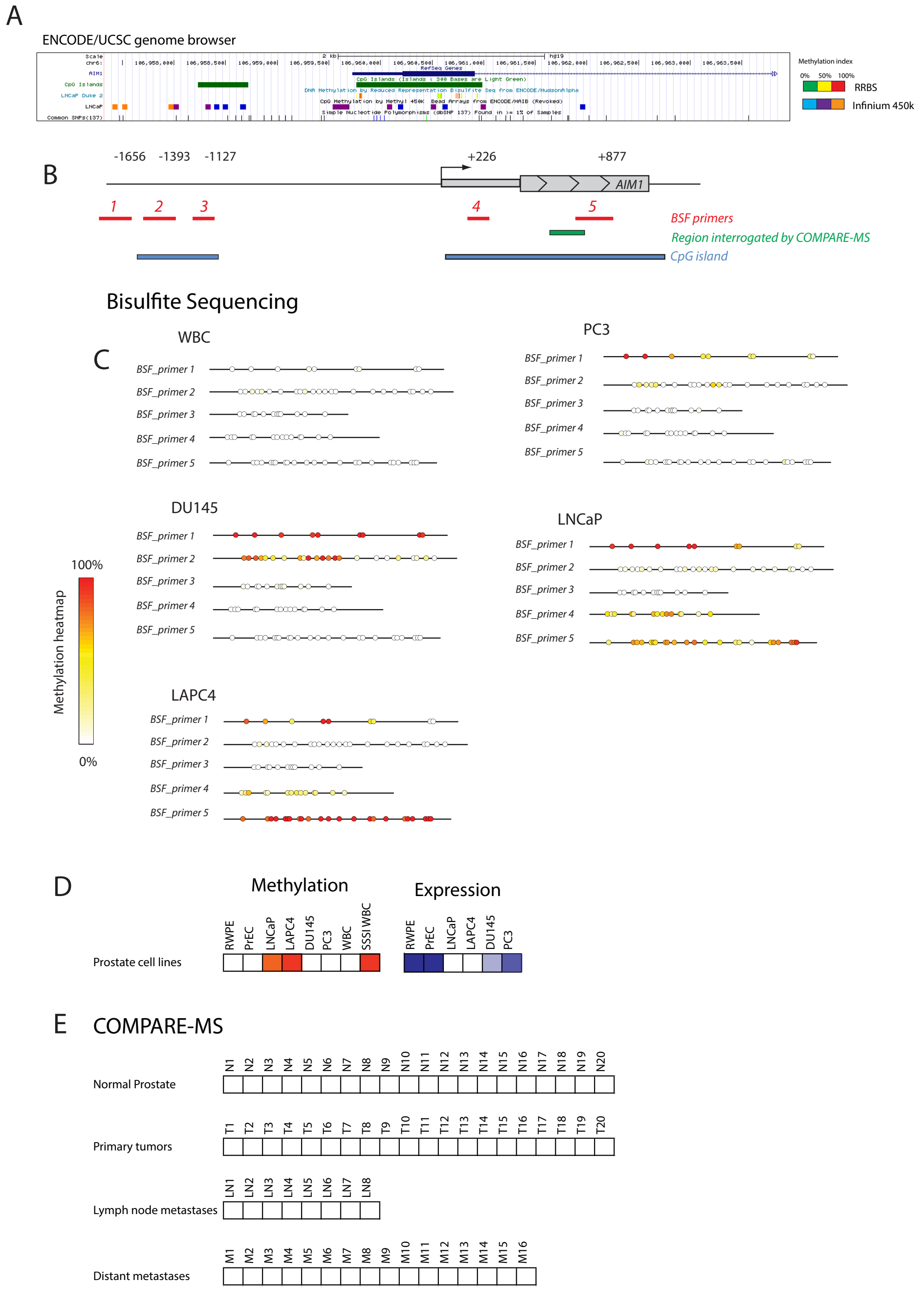

Supplementary Figure 6. DNA Methylation status of the AIM1 gene locus. (A) Publically available genome scale methylation analysis (reduced representation bisulfite sequencing (RRBS) and Infinium 450K methylation array) suggest CpG island methylation of the promoter region of *AIM1* in LNCaP cells (UCSC genome browser, http://genome.ucsc.edu, accessed 12/2014). (B) Ideogram depicting the upstream regulatory region of *AIM1*. Amplicon locations for bisulfite sequencing (BSF) and COMPARE-MS methylation analyses are highlighted in red and green respectively. (B) BSF amplicons interrogating CpG islands upstream and around the transcriptional start site of *AIM1* were analyzed by Sanger sequencing as described previously (13). (C) Integrated quantitative methylation estimates based on sequencing results from at least 8 individual clones are shown for each amplicon and are color coded with white indicating no methylation and red indicating dense methylation of all sequenced alleles. (D) The CpG island methylation status of a region around the transcriptional start site of AIM1 (see panel B) was further investigated using COMPARE-MS, a technology that combines methylation specific enrichment and methylation sensitive restriction enzyme digestion (13). Normal white blood cell DNA (WBC) and M.SssI treated fully methylated white blood cell DNA (SSSI WBC) are shown as positive and negative controls, respectively and were used for normalization. *AIM1* mRNA expression levels in these cell lines (determined in **Supplementary Figure 5**) are also displayed in color scale to assess correlations with methylation. LNCaP and LAPC4 cells showed strong methylation and low *AIM1* mRNA expression, while the other prostate cell lines showed absence of methylation and high *AIM1* expression. (E) Despite the presence of dense promoter associated CpG island methylation in the prostate cancer cell lines LNCaP and LAPC4, 0/20 normal prostate tissues, 0/20 primary tumors and 0/24 nodal and distant metastases showed any evidence form *AIM1* CpG methylation at this region. All sample details have previously been published (14).

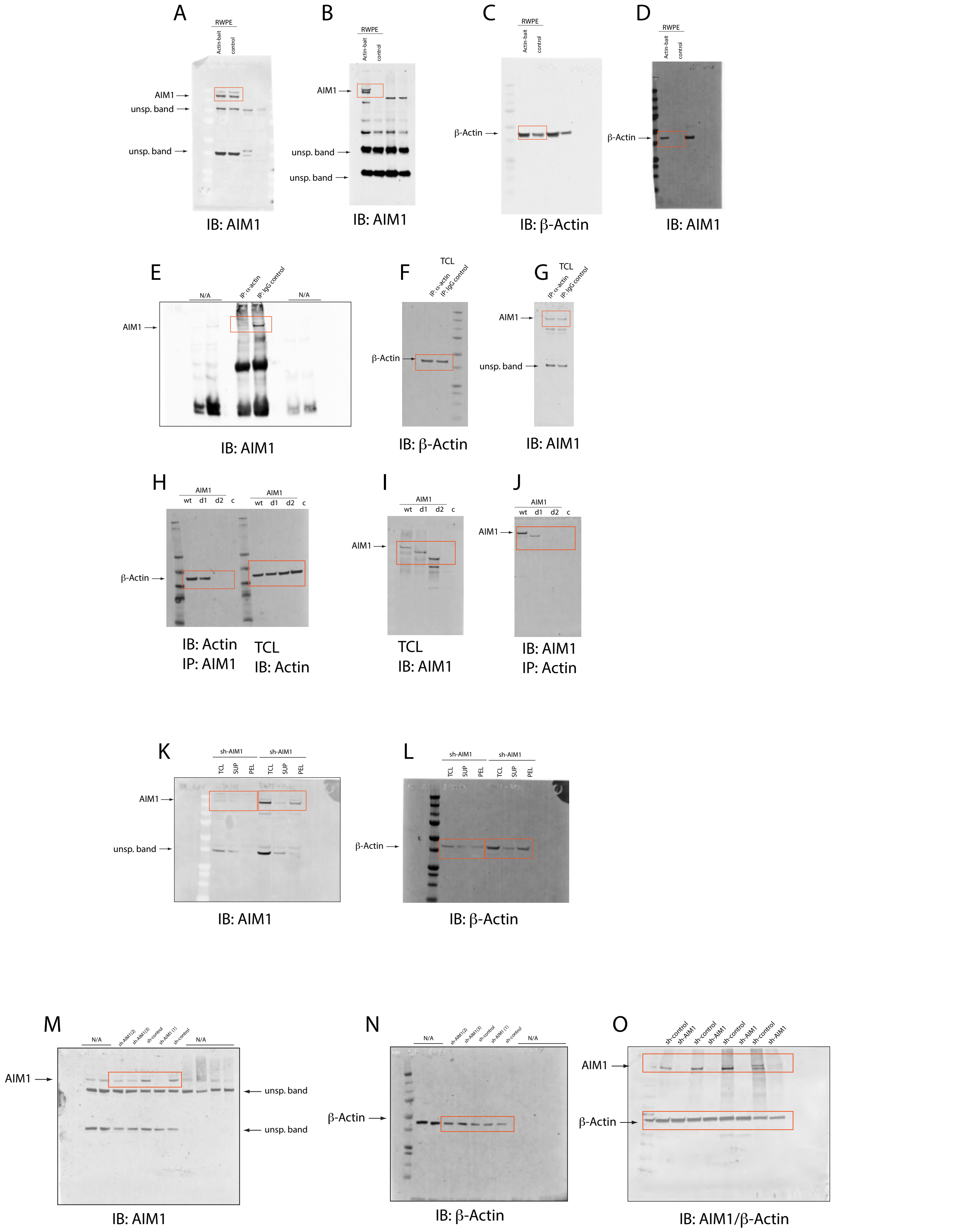

Supplementary Figure 7. Uncropped gels (A, B, C, D) Gel images of gels used in **Figure 1D**. (E, F, G) Gel images of gels used in **Figure 1E**. (H, I, J) Gel images of gels used in **Figure 2B**. (K, L) Gel images of gels used in **Figure 3D**. (M, N) Gel images of gels used in **Supplementary Figure 2.** (O) Gel image of gels shown in **Supplementary Figure 2**. . Note that cropped bands shown in figures are boxed in orange.

Supplementary Table 1. LC-MS/MS identification of proteins recovered after AIM1 pulldown in HEK293 cells.

			Raw counts		Normalized (Bait-control) counts
**Protein ID**	**Accession Number**	**MW**	**TAP_AIMI**	**TAP_CTR**	**Nor_AIM1_TAP**
absent in melanoma 1	*62988361*	189 kDa	*68*	*10*	**58**
myosin, heavy polypeptide 9, non-muscle	*12667788*	227 kDa	*34*	*7*	**27**
beta actin	*4501885*	42 kDa	*38*	*15*	**23**
myosin, heavy polypeptide 10, non-muscle	*41406064*	229 kDa	*33*	*16*	**17**
cardiac muscle alpha actin 1 proprotein	*4885049*	42 kDa	*23*	*7*	**16**
vimentin	*62414289*	54 kDa	*14*	*0*	**14**
keratin 2	*47132620*	65 kDa	*41*	*30*	**11**
heat shock 70kDa protein 1B	*167466173*	70 kDa	*18*	*9*	**9**
keratin 6A	*5031839*	60 kDa	*24*	*15*	**9**
keratin 1	*119395750*	66 kDa	*62*	*54*	**8**
clathrin heavy chain 1	*4758012*	192 kDa	*11*	*3*	**8**
drebrin 1 isoform a	*18426915*	71 kDa	*12*	*4*	**8**
keratin 5	*119395754*	62 kDa	*19*	*11*	**8**
keratin 10	*195972866*	59 kDa	*52*	*45*	**7**
nucleolin	*55956788*	77 kDa	*12*	*5*	**7**
filamin A, alpha isoform 1	*116063573*	280 kDa	*7*	*0*	**7**
poly (ADP-ribose) polymerase 1	*156523968*	113 kDa	*22*	*16*	**6**
tubulin, beta, 2	*5174735*	50 kDa	*11*	*5*	**6**
calmodulin 2	*4502549*	17 kDa	*7*	*2*	**5**
keratin 14	*15431310*	52 kDa	*13*	*9*	**4**
tubulin alpha 6	*14389309*	50 kDa	*9*	*5*	**4**
heat shock 90kDa protein 1, beta	*20149594*	83 kDa	*12*	*8*	**4**
coronin, actin binding protein	*7656991*	53 kDa	*5*	*1*	**4**
myosin, light chain 6	*17986258*	17 kDa	*6*	*2*	**4**
heat shock 70kDa protein 5	*16507237*	72 kDa	*7*	*3*	**4**
myosin IC isoform a	*124494238*	122 kDa	*4*	*0*	**4**
myosin VI	*92859701*	149 kDa	*4*	*0*	**4**
keratin 9	*55956899*	62 kDa	*31*	*28*	**3**
chaperonin	*31542947*	61 kDa	*13*	*10*	**3**
methylcrotonoyl-Coenzyme A carboxylase 2 (beta)	*11545863*	61 kDa	*5*	*2*	**3**
myosin regulatory light chain MRCL2	*15809016*	20 kDa	*6*	*3*	**3**
ribosomal protein L4	*16579885*	48 kDa	*3*	*0*	**3**
LIM domain and actin binding 1 isoform a	*165905589*	85 kDa	*3*	*0*	**3**
prohibitin 2	*6005854*	33 kDa	*4*	*1*	**3**
DEAH (Asp-Glu-Ala-His) box polypeptide 9	*100913206*	141 kDa	*3*	*0*	**3**
keratin 16	*24430192*	51 kDa	*12*	*9*	**3**
methylcrotonoyl-Coenzyme A carboxylase 1 (alpha) precursor	*116805327*	80 kDa	*3*	*0*	**3**
complement component 1	*4502491*	31 kDa	*4*	*1*	**3**
prohibitin	*4505773*	30 kDa	*3*	*0*	**3**
tyrosine 3/tryptophan 5 -monooxygenase activation protein	*4507953*	28 kDa	*4*	*1*	**3**
filamin B, beta (actin binding protein 278)	*105990514*	278 kDa	*3*	*0*	**3**
SERPINE1 mRNA binding protein 1 isoform 1	*66346679*	45 kDa	*3*	*0*	**3**
myosin IB isoform 1	*194328685*	132 kDa	*3*	*0*	**3**
protein phosphatase 1, regulatory subunit 9B	*140972063*	89 kDa	*3*	*0*	**3**
heat shock 70kDa protein 8 isoform 1	*5729877*	71 kDa	*11*	*9*	**2**
ribosomal protein L5	*14591909*	34 kDa	*5*	*3*	**2**
ribosomal protein P2	*4506671*	12 kDa	*5*	*3*	**2**
XTP3-transactivated protein A	*13129100*	19 kDa	*4*	*2*	**2**
pyruvate kinase, muscle isoform M1	*33286420*	58 kDa	*3*	*1*	**2**
ATP synthase, H+ transporting, mitochondrial F1 complex	*32189394*	57 kDa	*3*	*1*	**2**
heterogeneous nuclear ribonucleoprotein A1 isoform b	*14043070*	39 kDa	*4*	*2*	**2**
ribosomal protein L6	*16753227*	33 kDa	*3*	*1*	**2**
pyruvate carboxylase precursor	*106049292*	130 kDa	*2*	*0*	**2**
chaperonin containing TCP1, subunit 6A isoform a	*4502643*	58 kDa	*2*	*0*	**2**
heat shock protein 90kDa alpha (cytosolic), class A	*153792590*	98 kDa	*10*	*8*	**2**
eukaryotic translation elongation factor 1 gamma	*4503481*	50 kDa	*3*	*1*	**2**
chaperonin containing TCP1, subunit 3 isoform c	*58761484*	56 kDa	*3*	*1*	**2**
myosin ID	*51100974*	116 kDa	*2*	*0*	**2**
fatty acid synthase	*41872631*	273 kDa	*2*	*0*	**2**
desmoglein 2 preproprotein	*116534898*	122 kDa	*2*	*0*	**2**
nucleophosmin 1 isoform 1	*10835063*	33 kDa	*2*	*0*	**2**
ribosomal protein S3a	*4506723*	30 kDa	*2*	*0*	**2**
GNAS complex locus isoform f	*117938762*	46 kDa	*2*	*0*	**2**
ATP-dependent DNA helicase II	*10863945*	83 kDa	*2*	*0*	**2**
PREDICTED: hypothetical protein	*113420837*	12 kDa	*2*	*0*	**2**
myosin phosphatase-Rho interacting protein isoform 2	*50980301*	117 kDa	*2*	*0*	**2**
propionyl Coenzyme A carboxylase, beta polypeptide	*119943100*	58 kDa	*8*	*7*	**1**
solute carrier family 25, member 5	*156071459*	33 kDa	*4*	*3*	**1**
tyrosine 3/tryptophan 5 -monooxygenase activation protein	*5803225*	29 kDa	*2*	*1*	**1**
chaperonin containing TCP1, subunit 7 isoform a	*5453607*	59 kDa	*2*	*1*	**1**
ribosomal protein L8	*4506663*	28 kDa	*2*	*1*	**1**
PRKC, apoptosis, WT1, regulator	*55769533*	37 kDa	*2*	*1*	**1**
B-cell receptor-associated protein 31	*32171186*	28 kDa	*2*	*1*	**1**
heterogeneous nuclear ribonucleoprotein A3	*34740329*	40 kDa	*2*	*1*	**1**
ubiquitin and ribosomal protein L40 precursor	*4507761*	15 kDa	*2*	*1*	**1**
splicing factor 3b, subunit 1 isoform 1	*54112117*	146 kDa	*2*	*1*	**1**

Supplementary Table 2:
H-scores of Membranous and Cytoplasmic AIM-1 Expression

	Normal Prostate	Prostate Cancer	Lymph Node Metastasis	P value*
Number of cases	**81**	**87**	**52**	
**Membranous H-Score**				0.0001
Mean (SD)	286 (33)	70 (71)	76 (67)	
Median (IQR)	300 (0)	40 (90)	57.5 (90)	
**Cytoplasmic H-Score**				0.0001
Mean (SD)	3 (13)	111 (75)	70 (60)	
Median (IQR)	0 (0)	130 (100)	50 (50)	

Abbreviations: SD, standard deviation; IQR, interquartile range.

* Kruskal-Wallis test.

Supplementary Table 3:
Post hoc Comparison Tests Between H-scores of Membranous and Cytoplasmic AIM-1 Expression

H-Score	P value*
**Membranous**	
Nontumor Prostate Vs. Prostate Cancer	< 0.001
Prostate Cancer Vs. Lymph Node Metastasis	0.45
Nontumor Prostate Vs. Lymph Node Metastasis	< 0.001
**Cytoplasmic**	
Nontumor Prostate Vs. Prostate Cancer	< 0.001
Prostate Cancer Vs. Lymph Node Metastasis	0.005
Nontumor Prostate Vs. Lymph Node Metastasis	< 0.001

* Wilcoxon rank-sum test

Supplementary Table 4:
H-scores of Membranous and Cytoplasmic AIM-1 Expression According to Gleason Score

	Gleason Score			
	≤ 6	7–8	≥9	P value*
**Membranous H-Score**				0.0001
Mean (SD)	235 (105)	125 (111)	131 (112)	
Median (IQR)	300 (70)	90 (220)	115 (170)	
**Cytoplasmic H-Score**				0.0001
Mean (SD)	36 (70)	65 (71)	75 (75)	
Median (IQR)	0 (40)	45 (130)	50 (50)	

Abbreviations: SD, standard deviation; IQR, interquartile range.

* Kruskal-Wallis test.
